# Neighboring Effect‐Initiated Supramolecular Nanocomplex with Sequential Infiltration as Irreversible Apoptosis Inducer for Synergetic Chemo‐Immunotherapy

**DOI:** 10.1002/advs.202402809

**Published:** 2024-08-13

**Authors:** Mengjie Ye, Junfeng Hu, Linlin Han, Hengbo Zhang, Peng Xue, Yuejun Kang, Shuang Bai, Zhigang Xu

**Affiliations:** ^1^ Key Laboratory of Luminescence Analysis and Molecular Sensing Ministry of Education School of Materials and Energy & Chongqing Engineering Research Center, for Micro‐Nano Biomedical Materials and Devices Southwest University Chongqing 400715 P. R. China; ^2^ Shaanxi Province Center for Regenerative Medicine and Surgery Engineering Research the First Affiliated Hospital of Xi'an Jiaotong University Xi'an 710061 China; ^3^ State Key Laboratory of Chemo/Biosensing and Chemometrics Hunan University Changsha 410082 P. R. China; ^4^ Yibin Academy of Southwest University Yibin 644000 China; ^5^ Key Laboratory of Laser Technology and Optoelectronic Functional Materials of Hainan Province College of Chemistry and Chemical Engineering Hainan Normal University Haikou 571158 China

**Keywords:** chemotherapy, DNA damage, immune checkpoint blockade therapy, neighboring effect, supramolecular nanocomplex

## Abstract

Chemotherapy‐based combination regimens are recommended as first‐line treatment for colorectal cancer. However, multidrug resistance (MDR) and limited drug infiltration in tumor microenvironment remain critical challenges. Herein, a pH/redox dual activated supramolecular DAS@CD‐OxPt (IV) nanoparticles (NPs) via host‐guest molecular recognition to achieve relay drugs delivery of active oxaliplatin (OxPt (IV)) and Src inhibitor dasatinib (DAS) between tumor cells is developed. DAS@CD‐OxPt (IV) NPs exhibit prolonged circulation in the blood and intra‐tumoral retention. Triggered by the endo/lysosome (pH 5.0), flexible DAS@CD‐OxPt (IV) NPs exhibited proton‐driven in situ assembly to form nanofiber in tumor cells. Dual chemotherapeutic agents released from DAS@CD‐OxPt (IV) NPs synergistically cause irreversible DNA damage by blocking p53‐mediated DNA repair. Supramolecular nanofibers can further serve as the “ammunition depot” to continuously release drugs from dying cells and transport them into neighboring tumor cells, leading to domino‐like cell death and enhanced immunogenicity. Furthermore, DAS@CD‐OxPt (IV) NPs combined with immune checkpoint blockade (ICB) therapy strikingly suppress CT26 tumor growth and pulmonary metastasis.

## Graphical abstract

Benefiting from the neighboring effect, the permeability and cytotoxicity of DAS@CD‐OxPt NPs can be amplified by a “catching fire” infected model. With this, tumor cells are damaged layer by layer in a sort of “peeling off onions”, forwarding chemotherapeutic drugs to deeply penetrate into the tumor and triggering widespread apoptosis relentlessly.

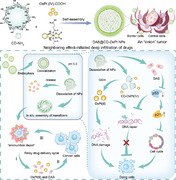

## 1 Introduction

Colorectal cancer (CRC) is the third most diagnosed cancer and the second most fatal cancer worldwide, posing a huge burden and threat to health. Currently, the treatment for CRC involves surgical resection, assisted by radiotherapy or chemotherapy.^[^ ^1^, ^2^, ^3^, ^4^ ^]^ Due to the presence of some unresectable tumors and recurrent and metastatic disease after surgery, chemotherapy is an essential treatment. Motivated by the demand for the safety and efficacy of chemotherapy, antineoplastic agents are doped into intelligent drug delivery systems to further promote the accumulation of drugs at tumor tissues for realizing an anticipated treatment.

A supramolecular drug delivery system directed by a flexible modular strategy has been proposed, and additional combinatorial advantages also arise from the modular flexible activation of supramolecular interactions.^[^ ^5^, ^6^, ^7^ ^]^ Supramolecular assembly utilizes the driving force between host and guest molecules to drive small molecule (pro)drugs into nanoassemblies, of which cyclodextrins (CDs) are widely known as host prodrugs. To date, many successful studies on minor modified CDs, CDs have been diffusely used as excipients in drug delivery systems to form supramolecular nanomedicine with hydrophobic chemotherapy drugs and promote solubility or stability of the drugs.^[^ ^8^, ^9^, ^10^, ^11^ ^]^ However, one major hindrance to therapeutic efficiency would be the difficulty in realizing deep infiltration in solid tumors due to the interference of the tumor microenvironment.

Benefiting from the neighboring effect, the permeability and cytotoxicity of chemotherapeutic nanomedicines can be amplified by a “catching fire” infected model.^[^ ^12^, ^13^, ^14^, ^15^ ^]^ Specifically, the nanomedicines are first internalized into the tumor cells, and the release of chemotherapeutic drugs will timely kill them. Subsequently, the drug‐loaded dying cells, like a spark, are treated as “pathogens” with the capability of “infection”, the drugs can be liberated from “pathogens” to “infect” surrounding cells. With this, tumor cells are damaged layer by layer in a sort of “peeling off onions”, forwarding chemotherapeutic drugs to deeply penetrate the tumor and triggering widespread apoptosis relentlessly. Nevertheless, the neighboring effect is still plagued by the depletion of chemotherapeutic drugs and multidrug resistance (MDR), especially, the nuclear‐mediated DNA damage chemotherapeutical nanomedicines.^[^ ^16^, ^17^, ^18^, ^19^ ^]^ It is highly desirable to exploit a novel strategy to deal with MDR and amplified neighboring effects.

Oxaliplatin (OxPt), a classical platinum‐based chemotherapeutic agent used to cure CRC. The cytotoxicity of OxPt (II) is mainly attributed to its cross‐linking abilities with DNA strands and the induction of DNA damage response (DDR), while intrinsic and acquired MDR is common.^[^ ^20^, ^21^, ^22^, ^23^ ^]^ Evidence from preclinical research suggests that Src inhibitor was expected to alter sensitivity for various chemotherapeutics, including platinum‐based chemotherapy.^[^ ^24^, ^25^, ^26^ ^]^ Therefore, dasatinib (DAS) is selected as a Src inhibitor in synergy with OxPt (II), which prevents DNA damage repair, induces cell cycle arrest, and assists OxPt (II) to continuously cause DNA damage and overcome MDR to a certain extent.

**Scheme 1:**
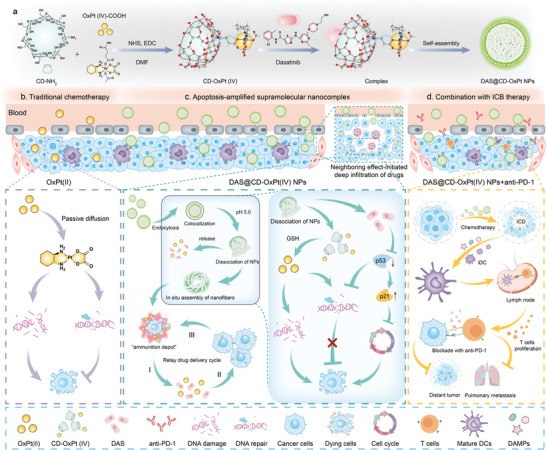
a) Schematic diagram illustrating the design of the supramolecular nanocomplex DAS@CD‐OxPt (IV) NPs for CRC chemo‐immunotherapy. b) Feasible mechanism of action in traditional OxPt (II). c) Mechanism of action in supramolecular nanocomplex as an inducer of irreversible apoptosis. I) In situ assembly nanofibers as “ammunition depot” through sustainable drug output. II) The domino‐like killing of neighboring tumor cells. III) Apoptosis‐amplified strategy through a relay drug delivery system. d) Synergistic effect and chemo‐immunotherapy in combination with anti‐PD‐1 antibody.

Herein, we raise a rational and effective designed precept for constructing a morphologically transformable supramolecular nano‐assembly, which is composed of tailored platinum (IV)‐conjugated CDs prodrug and dasatinib, driven by supramolecular recognition between them (designated as DAS@CD‐OxPt (IV) NPs). The supramolecular integration of DAS@CD‐OxPt (IV) NPs is endowed with chemotherapeutic drugs with desirable circulation of the blood and efficient accumulation at the tumor tissues. More interestingly, after arriving in tumor cells, this dynamic DAS@CD‐OxPt (IV) NPs exhibits a disassembly‐assembly modality due to the proton‐driven intrinsically flexible supramolecular interaction under acid stimulation, resulting in morphological transformation to spatially spiral nanofibers through in situ assembly. During this transformation, DAS molecules are released from DAS@CD‐OxPt (IV) NPs due to the protonation of the CDs cavity in acid endosomal/lysosomal microenvironment. Meanwhile, toxic OxPt (II) from CD‐OxPt (IV) conjugation is further activated by intracellular high glutathione (GSH) in tumor cells. Released DAS can synergistically induce robust and irreversible DNA damage caused by OxPt (II), resulting apoptosis through blocking p53‐mediated DNA repair and arresting the cell cycle. Moreover, the reassembled supramolecular nanofibers can become the “ammunition depot”, which are continuously liberated from dying/dead cells and transported into neighboring tumor cells. Eventually, this domino‐like drug penetration and apoptosis induction lead to the entire tumor collapse. Such a “relay race” collaborative therapy can surmount the limitations originating from neighboring effects and rely on sequential infiltration of drugs to activate the immune response and enhance immune checkpoint blockade (ICB) immunotherapeutic effect (**Scheme** **1**). Overall, the supramolecular inclusion mediated by neighboring effect can achieve continuous drug penetration to overcome MDR, facilitating the activation and infiltration of various immune cells in the center of tumor tissues, which provides potential translation for treating human cancers to simultaneously augment chemo‐immunotherapy effects.

## 2 Results and Discussion

### 2.1 Design and Construction of Host‐Guest Complexation DAS@CD‐OxPt (IV) NPs

**Figure 1:**
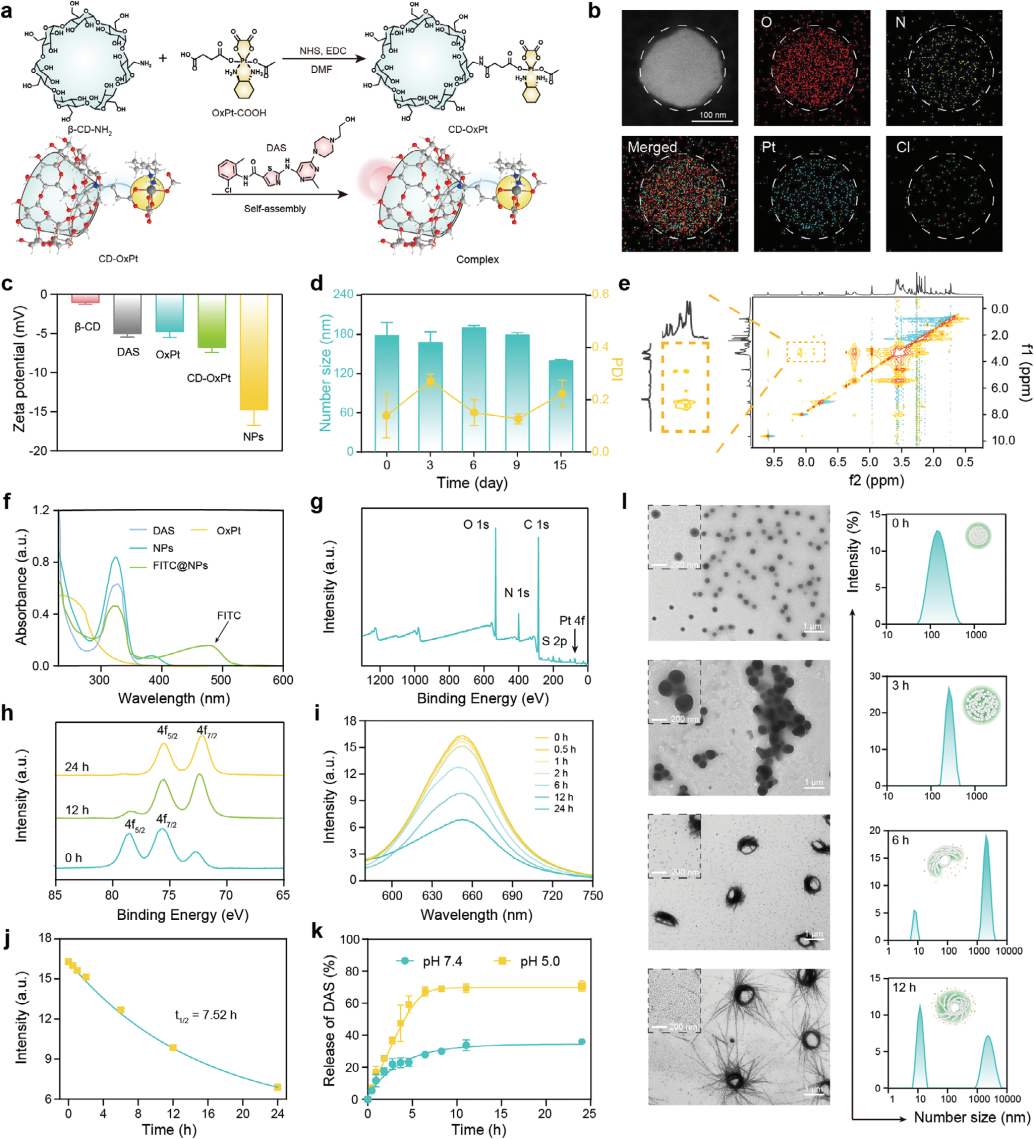
Synthesis and characterization of DAS@CD‐OxPt (IV) NPs. a) Synthetic route of CD‐OxPt (IV) and the host‐guest complexation process of DAS@CD‐OxPt (IV) complex. b) Scanning TEM elemental mapping of DAS@CD‐OxPt (IV) NPs (O, N, Pt, and Cl elements). c) The zeta potential of β‐CD, DAS, OxPt (II), CD‐OxPt (IV), and DAS@CD‐OxPt (IV) NPs dispersed in PBS. Data are presented as mean ± SD (*n* = 3). d) Stability of hydrodynamic diameter about DAS@CD‐OxPt (IV) NPs in a 15‐day observation period (*n* = 3). e) 2D‐NOESY NMR spectrum of the DAS@CD‐OxPt (IV) NPs in DMSO‐*d_6_*. f) UV–Vis spectra of free DAS, OxPt (II), DAS@CD‐OxPt (IV) NPs, and FITC‐labeled DAS@CD‐OxPt (IV) NPs (FITC@NPs) in PBS. g) XPS spectra of DAS@CD‐OxPt (IV) NPs. h) XPS curves of Pt in DAS@CD‐OxPt (IV) NPs after incubation with 10 mM GSH for 0, 12, 24 h. i) The fluorescence changes of the Nile Red‐labeled DAS@CD‐OxPt (IV) NPs (Nile Red@NPs) in the presence of 10 mM GSH (λex = 580 nm, λem = 750 nm). j) The fluorescence intensity at 652 nm was performed in a nonlinear manner and the fitted curve calculated the dissociation half‐life period of Nile Red@NPs with 10 mM GSH treatment. k) Controlled release profiles of DAS@CD‐OxPt (IV) NPs under pH 7.4 and 5.0 (*n* = 3). l) TEM images and relevant size distribution dissociation traces of DAS@CD‐OxPt (IV) NPs after exposure to pH 5.0 at 0, 3, 6, and 12 h.

The supramolecular prodrug of CD‐OxPt (IV) was synthesized via Bloch's reaction of OxPt (IV)‐COOH with mono‐(6‐amino‐6‐deoxy)‐β‐cyclodextrin (β‐CD‐NH_2_) (Figure S1, Supporting Information). The successful preparation of OxPt (IV)‐COOH and host monomers CD‐OxPt (IV) were confirmed by ^1^H nuclear magnetic resonance (NMR) and the high‐resolution mass (HRM) spectroscopies (Figures S2–S4, Supporting Information). The β‐CD possesses a more expansive chemical design space, and the CD‐OxPt (IV) with minor chemical modification of β‐CD can serve as a platform compatible with DAS. On account of the host‐guest molecular recognition motifs, CD‐OxPt (IV) encapsulates DAS within its cavity to form the interlocked molecule DAS@CD‐OxPt (IV) complex (**Figure** **1**a). Whereafter, interlocked molecule DAS@CD‐OxPt (IV) complex exhibits a higher order self‐assembly and forms sphere supramolecular nanocomplex (DAS@CD‐OxPt (IV) NPs). Based on a large number of host‐guest studies,^[^ ^27^, ^28^, ^29^ ^]^ the different molar ratio ratios were used to explore the optimal ratio between the guest molecule and the host molecule by comparing hydrodynamic diameter and polydispersity index. This host‐guest complexation started from optimizing the reaction molar ratio for constructing DAS@CD‐OxPt (IV) NPs, where molar ratio 1:1 was the optimal reaction condition, was used to study further, to attain significantly excellent hydrodynamic diameter (number size = 179.0 ± 19.3 nm), less polydispersity index (PDI = 0.211 ± 0.037) and lower zeta potential (zeta potential =−14.77 ± 0.79) (Figure S5, Supporting Information). The elemental mapping images of host‐guest complexation DAS@CD‐OxPt (IV) NPs (Figure 1b; Figure S6, Supporting Information) suggested the homogeneous distribution of each element (O, N, Pt, and Cl elements) on the core of DAS@CD‐OxPt (IV) NPs, verifying the formation of the supramolecular nanocomplex. By dispersing the different components in PBS, the zeta potential of DAS@CD‐OxPt (IV) NPs was significantly lower than that of the individual free components, which can be observed clearly, revealing the distinct advantages in blood circulation (Figure 1c). As exhibited in Figure 1d, the DAS@CD‐OxPt (IV) NPs were dispersed in PBS, and the supramolecular nano complex showed excellent stability and monodispersity over the 15‐day observation period.

The cross‐peak symmetry signals of 2D‐NOESY spectrum between DAS (7.0–10 ppm) and CD‐OxPt (IV) (2.5–4.0 ppm) revealed that H in the benzene ring of DAS were well correlated with internal protons H in the cavity of CD‐OxPt (IV), confirming the formation of inclusion complex DAS@CD‐OxPt (IV) NPs (Figure 1e). Meanwhile, the UV‐vis spectra further reinforced this perspective by the clear observation of the characteristic peak of DAS at 320 nm (Figure 1f). Considering that a beingless fluorescent signal was present for DAS and OxPt (II) in subsequent cell experiments, FITC was loaded for a series of cell experiments, including uptake, colocalization assay, and performance of penetration assay.

On account of the characteristic peak of OxPt was not directly observed by UV‐vis absorption, X‐ray photoelectron spectroscopy (XPS) was conducted to verify the presence of OxPt (IV) (Figure 1g). Given that OxPt (II) rather OxPt (IV) act as the ultimate DNA crosslinking agent, OxPt (IV) in the host‐guest complex of DAS@CD‐OxPt (IV) NPs was requested to be activated into OxPt (II) to result in inhibition of DNA replication and eventual tumor cells apoptosis and death,^[^ ^30^ ^]^ our arranged DAS@CD‐OxPt (IV) NPs supplied a double‐gated strategy (intracellular acid‐sensitive and GSH‐sensitive supramolecular nanocomplex), which was of significant importance in minimizing the systemic toxicity of OxPt (II). Here, the source of reduction‐sensitive release was the chemical nature of OxPt (IV). In higher GSH, the Pt‐O bond in CD‐OxPt (IV) would be broken, and the valence state transition of OxPt (IV) into OxPt (II) was verified by XPS. As shown in Figure 1h, two binding energy peaks of OxPt (IV) in DAS@CD‐OxPt (IV) NPs at 79.58 and 75.58 eV, OxPt (IV) were transformed into OxPt (II) (binding energies for Pt_4f_, 75.48 and 72.38 eV) after incubation with 10 mM GSH for 12 h. Meanwhile, the GSH‐responsiveness of DAS@CD‐OxPt (IV) NPs in a double‐gated strategy was further confirmed by fluorescence dissociation, in which the degradation of Nile Red@NPs in the presence of 10 mM GSH was studied at a prescheduled time. The result showed that DAS@CD‐OxPt (IV) NPs were able to deplete GSH, the dissociated Nile red was exposed to water, and fluorescence intensity gradually vanished, resulting in the dissociation half‐life period of 7.52 h (Figure 1i,j).

The host‐guest interaction derived from the unparalleled affinity between the cyclodextrin cavity receptor of CD‐OxPt (IV) and DAS guest element is considered the predominant reason for forming an intellective supramolecular nanocomplex. The presence of an acidic microenvironment in tumor cells for the realization of the controllable release of the supramolecular gated complex in an irritative responsive manner.^[^ ^31^ ^]^ On the premise that DAS@CD‐OxPt (IV) NPs can stably exist in vitro and circulate normally in the blood, the release modalities of DAS@CD‐OxPt (IV) NPs triggered by simulating the acidic tumor cells microenvironment were verified. The drug release study displayed that the DAS was released rapidly within 6 h, and the accumulative DAS release reached ≈70%. However, the lower release of drugs was detected at pH 7.4 (Figure 1k). The corresponding morphology images and relevant size distribution dissociation of DAS@CD‐OxPt (IV) NPs exposed to pH 5.0 were precisely observed by TEM and DLS analysis (Figure 1l). The slightly larger hydrodynamic diameter was detected by DLS could put down to the hydration layer around the DAS@CD‐OxPt (IV) NPs in aqueous solution, while TEM measurements would encounter dehydration resulting in size shrinkage of DAS@CD‐OxPt (IV) NPs. When DAS@CD‐OxPt (IV) NPs were exposed to an acidic solution, the supramolecular nanocomplex rapidly swelled within 3 h, and the supramolecular nanocomplex observed by TEM was ≈300 nm, which might be due to the protonation of the cavity in β‐CD and the weakening of host‐guest forces in the acidic environment. Subsequently, the supramolecular nanocomplex continued to collapse, and many of the larger fibrous aggregates were observed while small drug molecules with a particle size of ≈10 nm also appeared at 6 h. After 12 h incubation with a buffer solution at pH 5.0, the DAS@CD‐OxPt (IV) NPs completely disintegrated and the small molecule drug was widely distributed throughout the TEM. The accumulation of small molecule drugs could be explained by the fact that DAS@CD‐OxPt (IV) NPs were exposed to an acidic environment, the host‐guest forces continued to weaken, which could facilitate the drug diffusion across tumor tissue and improve therapeutic efficacy.

### 2.2 Driving Forces of Host‐Guest Complexation in DAS@CD‐OxPt (IV)

The binding driving forces of supramolecular assemblies are predominantly noncovalent, containing electrostatic interaction, π‐π stacking interaction, hydrophobic effects, van der Waals interaction, and hydrogen‐bonding interaction.^[^ ^32^ ^]^ The ordered supramolecular nanoarchitecture of specific shapes and sizes can effectively integrate by matching pieces due to binding driving forces.^[^ ^33^, ^34^ ^]^ After it was clear that the tumor cell environmental stimulation can achieve controllable release of a supramolecular gated complex, interactions as an essential player in supramolecular chemistry are worthy of deeper exploration. The associated atomic sequence number of CD‐OxPt (IV) and DAS is shown in Figure S7, Supporting Information.

**Figure 2:**
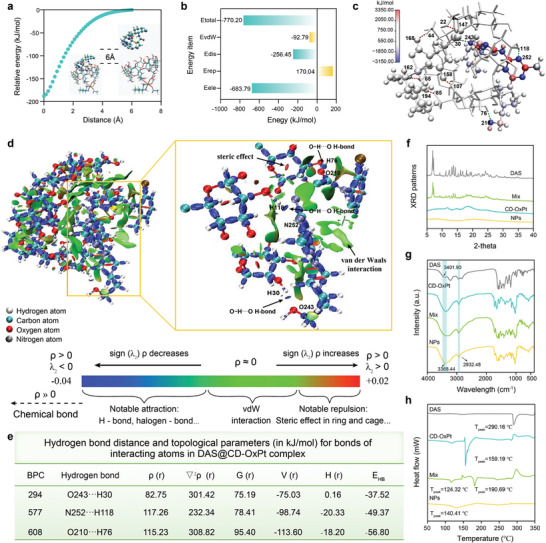
Driving forces of host‐guest complexation in DAS@CD‐OxPt (IV) NPs. a) Relative energy in the inclusion complexation of DAS molecule into CD‐OxPt (IV) molecule at different positions. b) and c) Decomposition of the total energetic contribution for DAS@CD‐OxPt (IV) complex and atomic coloring diagrams of intermolecular hydrogen bond (as shown in green dotted line) for DAS@CD‐OxPt (IV) inclusion complex and intramolecular hydrogen bond (as shown in red dotted line) formed by OxPt group and CD in CD‐OxPt (IV) molecule. d) Isosurface map of IRI = 0.8 for the DAS@CD‐OxPt (IV) inclusion complex. e) Topological parameters (kJ/mol) for bonds of interacting atoms of DAS@CD‐OxPt (IV) complex. (BCP—bond critical point; ρ(*r*)—electron density; ^∇2ρ(^ *^r^* ^)^—Laplacian of electron density; G(*r*)—electron kinetic energy density; V(*r*)—electron potential energy density, H(*r*)—total electron energy density; E_HB_—hydrogen bond energy). f) XRD patterns of DAS, CD‐OxPt (IV), Mix (molar ratio:1:1), and DAS@CD‐OxPt (IV) NPs. g) FTIR spectra of DAS, CD‐OxPt (IV), Mix, and DAS@CD‐OxPt (IV) NPs. h) DSC curves of DAS, CD‐OxPt (IV), Mix, and DAS@CD‐OxPt (IV) NPs.

As could be seen from **Figure** **2**a, the relative energy of the DAS@CD‐OxPt (IV) complex at the initial position was 0 kJ mol^−1^, and the relative energy of the DAS@CD‐OxPt (IV) complex was decreased as DAS gradually approached the CD‐OxPt (IV) cavity. When the moving distance of DAS was 6 Å, the energy of the DAS@CD‐OxPt (IV) complex was the lowest, which was −188.01 kJ mol^−1^, indicating that the molecular distance between DAS and CD‐OxPt (IV) was 0 Å, corresponding to the most stable conformation of the complex, which would be used for subsequent studies on the interaction between DAS and CD‐OxPt (IV).

According to the results of formula calculation and energy decomposition, the binding energies of DAS and CD‐OxPt (IV) complex were −92.79 and −770.20 kJ mol^−1^, respectively, both of which were negative, and were beneficial to the formation of the DAS@CD‐OxPt (IV) complex. The absolute value of binding energy calculated by density functional theory was quite different from that by EDA‐FF analysis, mainly due to the difference in calculation accuracy between density functional theory and molecular force field.

The weak interaction between molecules of the complex was quantitatively analyzed by EDA‐FF. The electrostatic interaction energy, repulsive interaction energy, and dispersive interaction energy of DAS@CD‐OxPt (IV) complex were −683.79, 170.04, and −256.45 kJ mol^−1^, respectively (Figure 2b). In the binding energy (−770.20 kJ mol^−1^) of the DAS@CD‐OxPt (IV) complex, on account of the presence of hydrogen bonds, electrostatic energy (−683.79 kJ mol^−1^) dominated, indicating that electrostatic force played a dominant role and hydrogen bond and van der Waals interactions had positive effects on the stability of the complex the electrostatic energy.

Subsequently, in order to display the specific interaction types and intensity between specific fragments, the results of the EDA‐FF analysis were atomically colored in the visualization software VMD, as shown in Figure 2c. The blue atoms indicated attraction, the red atoms indicated repulsive force, and the darker the color, the more pronounced the attraction/repulsive force, the white atoms were faintly contributing. Three intermolecular hydrogen bonds were formed between DAS and CD‐OxPt (IV) in the DAS@CD‐OxPt (IV) complex, were O210⋅⋅⋅H76, O243⋅⋅⋅H30 and N252⋅⋅⋅H118, separately. The acceptor oxygen (O) or nitrogen (N) atoms of intermolecular hydrogen bonds were all blue, showing that the formation of intermolecular hydrogen bonds contributed significantly to the stability of DAS@CD‐OxPt (IV) complex structures. Furthermore, the OxPt group five intramolecular hydrogen bonds were formed with the β‐CD parent, which was O22⋅⋅⋅H147, O85⋅⋅⋅H194, O158⋅⋅⋅H107, O162⋅⋅⋅H65, and O168⋅⋅⋅H44, separately. The existence of these hydrogen bonds was conducive to the stability of CD‐OxPt (IV) complexation.

The isoline of IRI analysis of the DAS@CD‐OxPt (IV) complex was exhibited in Figure 2d. The region of the red wafer was mostly located in the D‐pyranose ring of the CD‐OxPt (IV) molecule and the five and six rings of DAS, indicating that there was a steric effect in this region, and most areas of the isosurface were green, indicating that van der Waals interactions made a significant contribution in the interaction between CD‐OxPt (IV) and DAS. In addition, the intermolecular hydrogen bonds (blue regions) were formed between OH of C_2_ and C_3_ positions in parent β‐CD of CD‐OxPt (IV), and N and O atoms of DAS could be clearly observed, O210⋅⋅⋅H76, O243⋅⋅⋅H30 and N252⋅⋅⋅H118, respectively, which was consistent with the results observed by EDA‐FF.

The critical point topological parameters of the main hydrogen bond interactions calculated by the RI‐B3LYP‐D3(BJ)/def2‐TZVP level were mainly for the purpose of better reflecting the interaction strength of DAS@CD‐OxPt (IV) complex and obtaining the relevant the information of hydrogen bonds. The ∇^2^ρ(*r*) values of the primary hydrogen bond interaction critical point in the DAS@CD‐OxPt (IV) complex were positive, indicating that non‐covalent interaction (including hydrogen bonding and van der Waals interaction) was the main affinity in intermolecular interaction. H_(r)_ < 0, the noncovalent interaction was manifested as electrostatic interactions dominated by hydrogen bonds; H_(r)_ > 0, the non‐covalent interaction was represented by van der Waals interactions. H_(r)_ values of critical points 577 and 608 were negative, and the remaining H(r) values of the critical points were positive. Based on the classification of hydrogen bond strength according to Rozas et al.^[^ ^35^ ^]^ The N252⋅⋅⋅ H118 and O210⋅⋅⋅ H76 corresponding to critical points 577 and 608 respectively were moderate hydrogen bonds, and the intermolecular hydrogen bonds corresponding to the other critical points were weak hydrogen bonds, which were consistent with the consequence of EDA‐FF. In addition, among these weak hydrogen bonds, the most potent interaction was the critical point 294 (O243⋅⋅⋅H30), which was the largest ρ(r) (82.75 kJ mol^−1^) and the lowest bond energy (−37.52 kJ mol^−1^). The above analysis indicated that the interactions were dominated by stronger hydrogen bonds in the DAS@CD‐OxPt (IV) complex, followed by van der Waals interactions, which was in compliance with the results of EDA‐FF (Figure 2e; Table S1, Supporting Information). The DAS embedded into the β‐CD cavity rather than the straightforward physical mixture was confirmed by XRD diffractograms in which DAS and mixture revealed distinct peaks in the native form, DAS@CD‐OxPt (IV) NPs displayed a typical amorphous pattern (Figure 2f). In addition, the FT‐IR spectra of DAS, CD‐OxPt (IV), Mix and DAS@CD‐OxPt (IV) NPs were performed and shown in Figure 2g, β‐CD existed in all components demonstrated the characteristic absorption peaks with stretching vibration of O–H (3368.44 cm^−1^), C–H (2932.48 cm^−1^), the hydroxyl group (–OH) of DAS appeared at 3401.90 cm^−1^ could be observed in Mix. In contrast, the characteristic peak was not found in the DAS@CD‐OxPt (IV) NPs, which confirmed the inclusion formation. Ascribing to host‐guest complexation, the phase‐transition temperature (melting, boiling, or sublimating points) of the clathrate compound commonly shifted to distinct temperatures or disappeared. The DSC curves of DAS, CD‐OxPt (IV), Mix, and DAS@CD‐OxPt (IV) NPs exhibited a broad endothermic peak between 290.16 °C, 159.19, 124.32, and 140.41 °C, respectively, the weak phase transition temperatures between physical mixtures and clathrates corresponding to reasonable conclusion Niu et al. and Xi et al.^[^ ^36^, ^37^ ^]^ Thermal stability of DAS, CD‐OxPt (IV), Mix, and DAS@CD‐OxPt (IV) NPs by thermogravimetric analysis (TGA) also proved this aspect (Figure S8, Supporting Information).

### 2.3 In Vitro Potential Antitumor Evaluation of DAS@CD‐OxPt (IV) NPs

**Figure 3:**
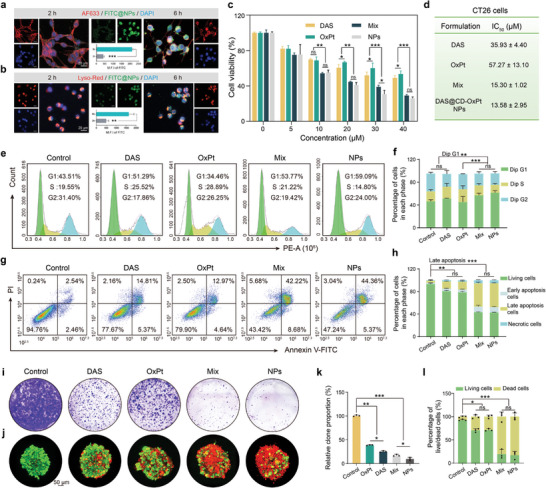
Synergistic in vitro toxicity assessment of DAS@CD‐OxPt (IV) NPs. a) Internalization and b) colocalization of lysosomes representative CLSM images in the CT26 cells cultured with FITC@NPs. (Blue fluorescence indicated nuclear staining with DAPI; AF633 and Lyso‐Red red fluorescence indicated cytoskeleton and lysosome; green fluorescence presented the internalization of NPs) Scale bars = 20 µm. c) Cell viability of CT26 was determined using the MTT assay after 24 h incubation with DAS, OxPt (II), Mix, and DAS@CD‐OxPt (IV) NPs (*n* = 3). d) IC_50_ of formulation in different treatment groups of CT26 cells. e) Cell cycle and f) flow cytometry analysis results of CT26 cells incubation with PBS, DAS, OxPt (II), Mix, and DAS@CD‐OxPt (IV) NPs (10 µM, *n* = 3). g) Apoptosis of CT26 tumor cells and h) quantification after treatment with various formulations including free DAS, OxPt (II), Mix, and DAS@CD‐OxPt (IV) NPs (10 µM, *n* = 3). i) Colony formation assay and k) quantitative analysis of CT26 cells treated with free DAS, OxPt (II), Mix, and DAS@CD‐OxPt (IV) NPs (5 µM, *n* = 3). j) Live/Dead assay and l) quantitative analysis in 3D multicellular spheroids (MCSs) of CT26 cells treated with various drugs (40 µM, *n* = 3). The living cells were stained green by FDA and the dead cells were stained red by PI. Scale bar = 50 µm.

Efficient endocytosis is a decisive procedure to strengthen drug accumulation and the effective treatment of tumor cells. The cellular uptake and intracellular distribution of the DAS@CD‐OxPt (IV) NPs in CT26 cells were preferentially investigated by FCM and CLSM. The fluorescence intensity of FITC@NPs increased with longer treatment time and reached 89.89% at 6 h (Figure S9, Supporting Information). Subsequently, the significant accumulation of FITC@NPs on the nucleus was observed by CLSM, turning out that the NPs could be efficiently internalized (**Figure** **3**a,b). To explore the potential antitumor evaluation of DAS@CD‐OxPt (IV) NPs in vitro, the MTT assay of NPs was performed on CT26 tumor cell lines, the IC_50_ of DAS and OxPt (II) on CT26 were 35.93 ± 4.40 µM and 57.27 ± 13.10 µM, while the IC_50_ of Mix and NPs were 15.30 ± 1.02 µM and 13.58 ± 2.95 µM, demonstrating combined therapy of supramolecular complexation enhanced the therapeutic efficiency in CT26 cells (Figure 3c,d).

To further appraise whether the damage potential of the experimental group was connected with the cell cycle arrest or cell apoptosis in CT26 cells, some interrelated experiments were conducted. In the detected diagram of the cell cycle (Figure 3e,f), the DAS and OxPt (II) could effectively arrest cells in the G1 phase (51.29%) and S phase (28.89%), severally, which were mainly due to the different damage mechanisms in two drugs. As DAS was a TKI drug with various kinases, the basis of reports, verified that DAS could increase the expression of intracellular p53 and p21, downregulate the expression of cyclin D1, block the cell cycle in G1 phase, and inhibit the proliferation of cancer cells,^[^ ^38^ ^]^ while the cell arrest in S phase treatment with OxPt (II) was mainly due to DNA damage.^[^ ^39^ ^]^ The excellent cycle arrest was detected in DAS@CD‐OxPt (IV) NPs, which arrested cells in the G1 phase (59.09%). As shown in the apoptosis chart (Figure 3g,h), compared to the 14.81% or 12.97% apoptosis rate in the DAS and OxPt (II) group, the apoptosis rate of Mix and DAS@CD‐OxPt (IV) NPs reached the highest level of 42.22% and 44.36%, which were ≈3 times that of DAS and OxPt (II) group. The distribution of apoptosis observed by CLSM was consistent with that of flow analysis (Figure S10, Supporting Information), showing excellent toxicity of combination chemotherapy.

Furthermore, the colony formation assay was conducted to determine whether DAS@CD‐OxPt (IV) NPs would influence the proliferation potential of CT26 cells, and the 2D/3D live/dead assay was conducted to visually observe the proportion of live/dead cells. Surviving colonies of CT26 cells were displayed in Figure 3i,k, and lower cell colonies proportion were produced in DAS (24.90%) than OxPt (II) (38.55%), possibly due to DAS being a potent G1 cycle blocker that significantly inhibited the division and proliferation of CT26 cells, and not surprisingly, the lowest surviving colonies proportion was come up in DAS@CD‐OxPt (IV) NPs (9.74%). The 2D/3D live/dead results (Figure 3j,l; Figures S11, and S12, Supporting Information) showed that DAS@CD‐OxPt (IV) NPs resulted in more cell death compared to an individual formulation (DAS or OxPt (II)), basically consistent with the experimental results of apoptosis. It was noted that Mix and DAS@CD‐OxPt (IV) NPs at all treatments showed higher cytotoxicity to CT26 cells than free DAS or OxPt (II), revealing that combined chemotherapy‐mediated therapy.

### 2.4 Neighboring Effect and Immune Response of DAS@CD‐OxPt (IV) NPs In Vitro

**Figure 4:**
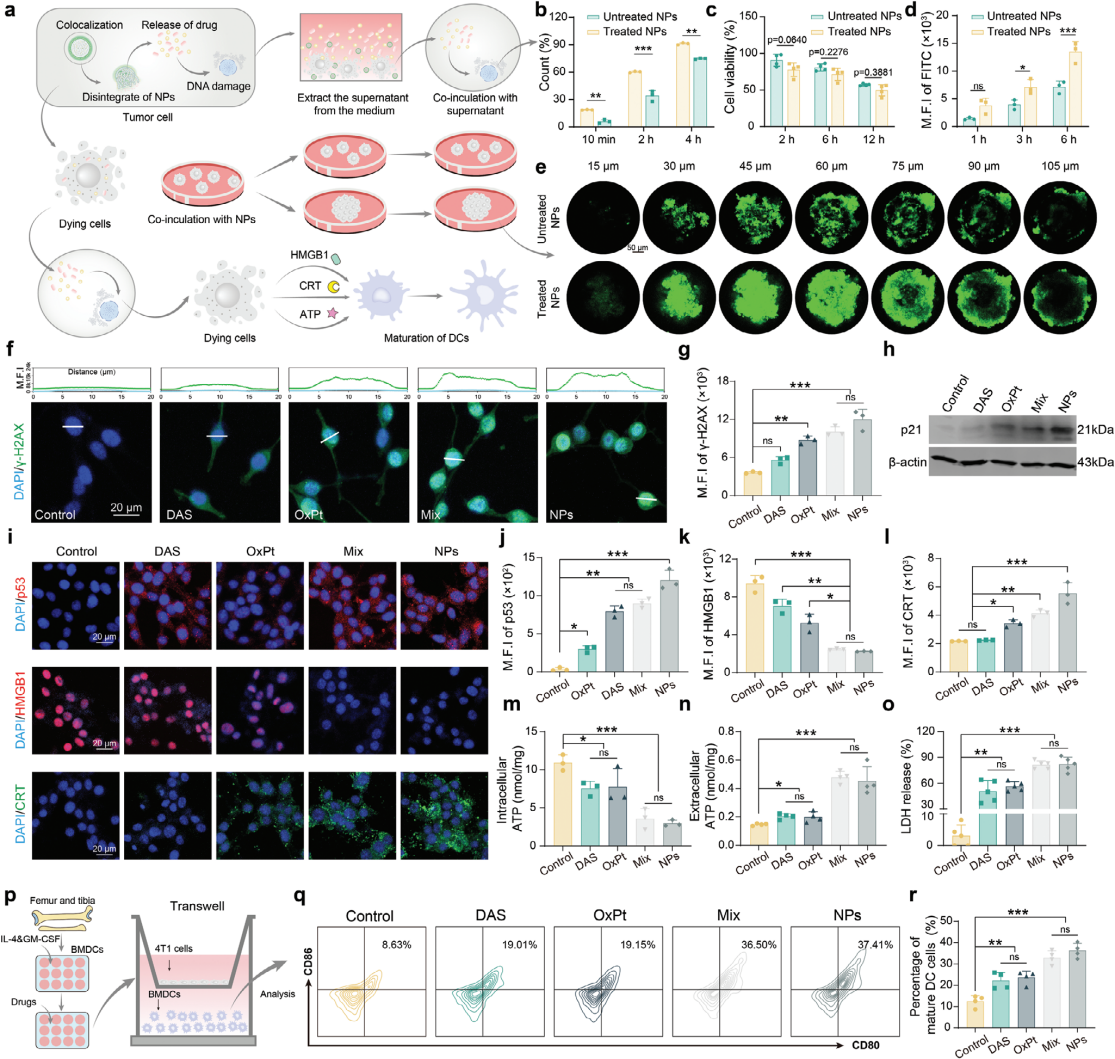
The neighboring effect and immunostimulatory activity of DAS@CD‐OxPt (IV) NPs in vitro. a) Schematic illustration of the experimental approach to verify the activation of neighboring effect and immune response. b) Cell uptake analysis of CT26 cells treated with DAS@CD‐OxPt (IV) NPs‐treated supernatants (the supernatants of DAS@CD‐OxPt (IV) NPs after 12 h incubation with CT26 cells) or untreated DAS@CD‐OxPt (IV) NPs. c) Cell viability assessment of CT26 cells treated with treated or untreated DAS@CD‐OxPt (IV) NPs. d) The permeability analysis of DAS@CD‐OxPt (IV) NPs into the MCSs after incubation with treated or untreated FITC@NPs. e) Scanning fluorescence imaging of cell layers at different depths in MCSs treated with DAS@CD‐OxPt (IV) NPs‐treated supernatants or untreated DAS@CD‐OxPt (IV) NPs for 3 h. Scale bar = 50 µm. f) Fluorescence images and g) quantification to show the DNA damage marker of γ‐H2AX in CT26 cells treated with PBS, DAS, OxPt (II), Mix, and DAS@CD‐OxPt (IV) NPs for 24 h (Blue fluorescence indicated nuclear staining with DAPI; green fluorescence indicated γ‐H2AX). Scale bar = 20 µm. h) The p21 proteins expression in CT26 cells with various treatments for 24 h by WB. i) CLSM images of p53 expression, CRT exposure, and HMGB1 release in CT26 cells treated with PBS, DAS, OxPt (II), Mix, and DAS@CD‐OxPt (IV) NPs for 24 h. Scale bar = 20 µm. j) Quantification analysis of p53, k) HMGB1, and l) CRT in CT26 cells treated with PBS, DAS, OxPt (II), Mix, and DAS@CD‐OxPt (IV) NPs. m) The intracellular (*n* = 3) ATP and n) extracellular (*n* = 4) ATP release in CT26 cells after various treatment (20 µM). o) The LDH release of CT26 cells after treatment with various formulations, including PBS, free DAS, OxPt (II), Mix, and DAS@CD‐OxPt (IV) NPs (40 µM, *n* = 5). p) Schematic illustration of the experimental approach to verify maturation of BMDCs. q) Flow cytometric data and corresponding proportion of DCs maturation in different groups. r) Quantity analysis of the flow cytometer on DCs maturation (*n* = 3, in the gate of CD11c^+^).

As shown in **Figure** **4**a, to confirm neighboring effect existed in DAS@CD‐OxPt (IV) NPs to release surplus drugs from apoptotic cells and then directly penetrate surrounding cells to induce cell death, fresh CT26 cells and MCSs were treated with the supernatant from DAS@CD‐OxPt (IV) NPs‐treated (treated NPs) cells. The cell uptake, cytotoxicity, and permeability analysis of DAS@CD‐OxPt (IV) NPs were conducted once more. In terms of cytophagocytosis (Figure 4b; Figure S13, Supporting Information), uptake of NPs‐treated cells was accelerated by 12.69%, 26.17%, and 16.35% relative to untreated NPs cells at 10 min, 2 h, 4 h, respectively, might be due to the treated NPs entered the cell mainly through osmosis. In subsequent toxicity experiments, although a particular cell activity gap was 12.77%, 9.36%, and 7.63% between the treated NPs and the untreated NPs at 2, 6, 12 h, there were no significant differences, regrettably (Figure 4c). Drug permeability analysis of MCSs confirmed enhanced penetrativity in treated NPs compared with untreated NPs (Figure 4d,e; Figures S14, and S15, Supporting Information).

It has been noted that nucleus‐mediated chemotherapy drugs, including platinum drugs and anthracyclines, could restrain DNA synthesis by giving rise to continual DNA single‐strand breaks, resulting in DNA damage, cycle arrest, and cell death.^[^ ^40^ ^]^ In addition, H2AX was phosphorylated to gamma‐H2ax by ataxia mutated gene (ATM) through a cascade reaction, and γ‐H2AX acted as a prominent marker of DNA damage.^[^ ^41^, ^42^ ^]^ In response to the DNA‐damaging course, replication stress was induced, and γ‐H2AX was activated. Cells communicate DNA damage signals to DNA repair kinases such as ATM, resulting in the degradation of p53.^[^ ^43^, ^44^ ^]^ To investigate the mechanism of DAS@CD‐OxPt (IV) NPs inducing DNA damage, not only the expression of γ‐H2AX but p53 was needed to detect in CT26 cells treated with different drugs. The more significant green fluorescence intensity of cells treated with DAS@CD‐OxPt (IV) NPs was observed compared with those treated with DAS or OxPt (II), while a puny significant difference was from Mix (Figure 4f,g). This might be due to the failure of DNA damage repair in NP‐treated cells, where DAS reversed the relative levels of p53 associated with DNA damage repair pathways, p53 and the downstream protein p21 were highly expressed. This conjecture was confirmed by WB analysis and CLSM pictures (Figure 4h,j).

As several platinum drugs are renowned for triggering cell death by immunogenic cell death (ICD), the ability of DAS@CD‐OxPt (IV) NPs to induce ICD was investigated by several typical biomarkers.^[^ ^45^ ^]^ The 3 typical hallmarks of ICD were significant surface exposure of calreticulin (CRT), elevated release of high‐mobility group box 1 (HMGB1), and secretion of adenosine triphosphate (ATP) into the extracellular milieu.^[^ ^46^ ^]^ Indeed, the maximal migration of the HMGB1 from the nucleus was observed in DAS@CD‐OxPt (IV) NPs and Mix by red fluorescence and quantitative analysis (Figure 4k). Similarly, upon treatment with DAS@CD‐OxPt (IV) NPs, markedly increased surface exposure of CRT and intracellular ATP transferred to the outside were monitored, demonstrating the potential of DAS@CD‐OxPt (IV) NPs for immune activation (Figure 4l–n). Lactate dehydrogenase (LDH) leakage further suggested that DAS@CD‐OxPt (IV) NPs could increase membrane permeability and favor the occurrence of the typical hallmarks (Figure 4o). Combined the secretion of ATP, surface exposure of CRT, and the release of HMBG1 indicated that treatment of CT26 cells with OxPt (II) triggered a firmer ICD than DAS, among the groups, DAS@CD‐OxPt (IV) NPs had the most robust ability to induce ICD.

Definitively, to confirm the immune activation effect of DAS@CD‐OxPt (IV) NPs as shown in Figure 4p, the maturity of DCs in treatment groups was detected, and bone marrow‐derived dendritic cells (BMDCs) were treated and the single cell was stained with CD11c, CD80, and CD86 antibodies. As shown in Figure 4q,r, the maturation of DC cells reached 37.41% in the co‐cultured systems, which was 4.33 times that of the control group, demonstrating that DAS@CD‐OxPt (IV) NPs could effectively promote the maturation of DC cells and produced the antitumor immune effect. The DAS synergized with OxPt (II) in DAS@CD‐OxPt (IV) NPs was conducive to enhancing ICD and in turn driving stronger immune responses.

### 2.5 In Vivo Fluorescence Imaging, Distribution and Antitumor Activity of DAS@CD‐OxPt (IV) NPs

**Figure 5:**
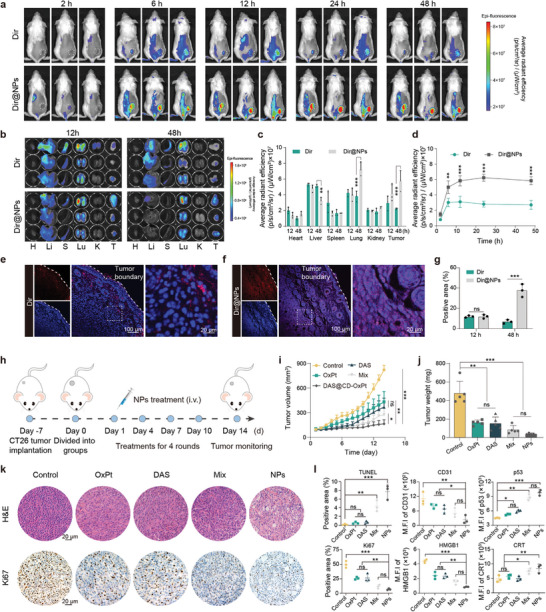
In vivo fluorescence imaging, distribution, and antitumor therapeutic potential efficiency. a) Representative fluorescence images of free Dir and Dir@NPs, in CT26 tumor‐bearing mice at expected times (*n* = 3). b) Ex vivo fluorescence images of major organs (H, heart; Li, liver; S, spleen; Lu, lung; K, kidney) and tumors (T, tumor) excised from mice at 12 and 48 h after being injected with Dir and Dir@NPs (*n* = 3). c) Average radiant efficiency of major organs and tumors were collected at 12 h and 48 h (*n* = 3). d) Quantification of average fluorescence efficiency at tumor tissues at different time points (*n* = 3). e), f) Fluorescence images of CT26 tumor tissues, and g) quantitative results of drug penetration after injected Dir and Dir@NPs at 48 h (blue, DAPI; red, Dir, *n* = 3). Scale bars = 100 µm. Zoom area = 20 µm. h) Schematic illustration of the in vivo therapeutic study. i) Tumor growth inhibition curves of average relative tumor volume during the therapeutic period (*n* = 5). j) Relative tumor weight of various treatment groups after the therapeutic period ended (*n* = 5). k) H&E and Ki67 histochemistry staining of excised tumor tissues after treatment ended (*n* = 3). Scale bars = 20 µm. l) Quantitative analysis from TUNEL, CD31, p53, Ki67, HMGB1, and CRT staining sections (*n* = 3).

Given the excellent performance of DAS@CD‐OxPt (IV) NPs in vitro, we further investigated biodistribution behaviors. The enriched fluorescence of free Dir at tumor sites was prominently less than that of Dir@NPs throughout the observation period. With a longer time after injection of Dir@NPs, the fluorescence signal of tumors increased continuously, reaching the peak of fluorescence at 24 h, the mean fluorescence intensity of tumors exhibiting Dir@NPs group was 2.22 times stronger than that of free Dir treated ones (**Figure** **5**a,d). The mice were euthanized 12 and 48 h after injection, and major organs removed from mice were observed. However, a lot of accumulated fluorescence of visceral organs in free Dir and Dir@NPs groups, mainly the liver, spleen, and lung at 12 h, Dir@NPs was barely retained in the heart, spleen, and kidney. Only a tiny portion of Dir@NPs remained in the liver at 48 h (Figure 5b,c). Attributed to the good systemic circulation ability, EPR effect, and enhanced penetration of Dir@NPs, the supramolecular nano complex tended to consistently remain in tumor tissue, and the fluorescence intensity of Dir@NPs was 2.16 times that of free Dir at tumor tissues. The histologic sections from removed tumors at 12 and 48 h were monitored with CLSM to verify the permeability of Dir@NPs. As shown in Figure 5e–g and Figure S16, Supporting Information, only weak fluorescence signals could be observed in both Dir and Dir@NPs at 12 h, and then the stronger fluorescence signals and significantly increased penetration depth were observed in the Dir@NPs group at 48 h, while fluorescence signals from Dir group existed in the surface layer of solid tumors. These results indicated that the size conversion of supramolecular nanocomplex induced by drug release conduced to enhance the neighboring effect and thus enhanced the penetration depth in the tumors.

Next, encouraged by reinforced cytotoxicity and effective accumulation at tumor sites of DAS@CD‐OxPt (IV) NPs, the antitumor activity of DAS@CD‐OxPt (IV) NPs was further investigated by following schedules (Figure 5h). The growth curves of tumors in mice treated with different treatments are shown in Figure 5i and Figure S17, Supporting Information, the treatments with OxPt (II) (tumor growth inhibition rate: 68.01%) and DAS (inhibition rate: 66.27%) exhibited a minor effect in inhibiting tumor growth, the inhibitory effect of DAS@CD‐OxPt (IV) NPs (inhibition rate: 92.05%) were obviously more significant than that of other treatments (Figure S18, Supporting Information). Also, Tumor weight was consistent with tumor suppression rate, and the superior tumor inhibition efficiency of DAS@CD‐OxPt (IV) NPs to other treatments, demonstrating the advantages of supramolecular inclusion (Figure 5j). More than halfway through the treatment, although Mix (inhibition rate: 82.09%) also had a relatively good therapeutic effect, serious side effects could be observed from a weight loss of ≈1.0 g in OxPt (II) and Mix groups due to the presence of OxPt (II) (Figure S19, Supporting Information). After cessation of injection, the body weight of mice with treatment increased steadily.

Finally, we verified the therapeutic effect of DAS@CD‐OxPt (IV) NPs by histopathological section analysis. The results showed that the negligible necrosis and massive tumor cell proliferation in the H&E and Ki67 tumor slices of the control group, while the treatment with DAS@CD‐OxPt (IV) NPs induced the most significant tumor cell necrosis, and proliferation inhibition, followed by the groups of Mix, free DAS, and free OxPt (II) (Figure 5k). During treatment, H&E staining results of major organs (heart, liver, spleen, lung, kidney) showed no histological damage (Figure S20, Supporting Information), indicating the excellent biosafety of DAS@CD‐OxPt (IV) NPs. Additionally, the quantitative analysis of apoptotic cells distribution in TUNEL, tumor angiogenesis in CD31, p53 expression (Figure S21, Supporting Information), the release of HMGB1, and exposure of CRT (Figure S22, Supporting Information), were basically consistent with the above and revealed that the DAS@CD‐OxPt (IV) NPs treatment could inhibit tumor growth in vivo via the chemotherapy medicated by multifunctional supramolecular inclusion (Figure 5l).

### 2.6 Chemo‐Immunotherapy of CT26‐Tumor‐Bearing Mice by Integrating DAS@CD‐OxPt (IV) NPs with anti‐PD‐1 Antibody

**Figure 6:**
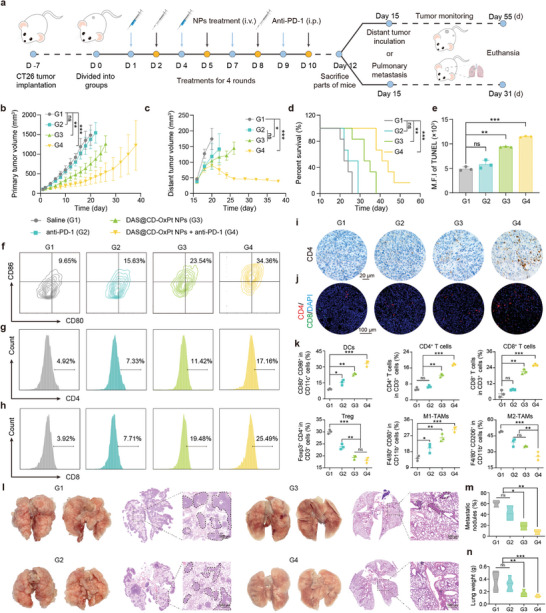
Therapeutic anticancer effect of combination therapy with anti‐PD‐1 antibody in the metastatic and survival CT26‐tumor‐bearing animal model. a) Schematic illustration of survival and pulmonary metastasis therapeutic strategy. b) Primary and c) distant tumor growth curves of combination therapy with anti‐PD‐1 antibody after different treatments (*n* = 6). d) Survival therapeutic curves in different treatment groups (*n* = 6). e) Quantitative analysis of TUNEL (*n* = 3). f) Representative flow cytometry plots indicating the proportions of DC maturation (in the gate of CD11c^+^). g) CD4^+^ and h) CD8^+^ T cells (*n* = 3, in the gate of CD3^+^) in lymph nodes after treatment ended. i) Immunohistochemistry staining CD4^+^ T cells in tumors after treatment ended, and j) immunofluorescent staining of CD4^+^ T cells and CD8^+^ T cells in tumors after treatment ended. k) The percentage of DCs, CD4^+^ T cells, CD8^+^T cells, Tregs (in the gate of CD3^+^), TAMs (in the gate of CD11b^+^ F4/80^+^) populations by flow cytometry (*n* = 3). l) Representative photographs and H&E staining sections of metastatic nodules in the lung in different groups after treatment. m) The percentage of metastatic nodules and n) weight of lung tissues after different treatments (*n* = 3).

The tumor microenvironment is the “Garden of Eden” where cancer cells live and grow, and it is composed not only of cancer cells but also of many different immune cells, that interact and communicate with the tumor microenvironment.^[^ ^47^, ^48^ ^]^ Hence, we investigated the DAS@CD‐OxPt (IV) NPs combined with anti‐PD‐1 antibodies to boost the function of related immune cells in immune responses. Exhibited in **Figure** **6**a, based on the model of antitumor activity, bilateral tumor models and lung metastasis models were constructed for the purpose of exploring the immune response efficacy and the abscopal effect in vivo.

As expected, the changing trend of tumor growth observed was similar to the results of tumor volume, and there was no significant change in the body weight of mice in each group throughout the treatment (Figure S23, Supporting Information). The tumors proliferated in control mice and anti‐PD‐1‐treated mice, while the reasonably efficient tumor suppression effect with the treatment of DAS@CD‐OxPt (IV) NPs (Figure 6b,c). This means that the bioavailability of drugs and immune activation were limited by a single treatment based on anti‐PD‐1 antibody and DAS@CD‐OxPt (IV) NPs. Hence, the DAS@CD‐OxPt (IV) NPs combined with anti‐PD‐1‐mediated immune checkpoint blockade (ICB) could carry out the most significant tumor suppression among all groups regardless of primary or distant tumors. In addition, the adoption of the combined strategy was able to significantly prolong the survival time of the mice (Figure 6d), and the relevant pathological tissue slices and mean fluorescence intensity after various treatments were consistent tumor suppression experimental results, including histopathology analysis of H&E, Ki67, and TUNEL, demonstrating the advantages of combination with anti‐PD‐1 antibody therapy (Figure 6e; Figure S24–S26, Supporting Information).

Indicators of infiltration and activation of immune cells were further examined, such as maturation of dendritic cells (DCs), activation of T cells, and phenotypic transformation of tumor‐associated macrophage (TAMs), the gating strategy is shown in Figure S27, Supporting Information. The highest DC maturation rate in the DAS@CD‐OxPt (IV) NPs + anti‐PD‐1 antibody treatment (34.36%) was significantly increased by ≈24% compared with that of the control group (Figure 6f), while the individual treatment with DAS@CD‐OxPt (IV) NPs only increased by ≈14% suggesting DAS@CD‐OxPt (IV) NPs was an effective immune inducer to boost systemic immunity. Meanwhile, CD4^+^ and CD8^+^ T cells, a major effector T lymphocyte to induce antitumor effects, were dramatically activated to infiltrate into tumors and were further explored. After treatment with DAS@CD‐OxPt (IV) NPs + anti‐PD‐1 antibody, the CD4^+^ and CD8^+^ T cells had the most remarkable tumor infiltration among all groups, the proportions of CD4^+^ and CD8^+^ T cells were 17.16% and 25.49% in lymph nodes, which were 3.25 times and 8.99 times higher than CD4^+^ and CD8^+^ T cells of the control group (Figure 6g,h). Similarly, the immunohistochemistry and immunofluorescence sections of CD4^+^ and CD8^+^ T cells in primary tumors were visually monitored under the fluorescence microscope and CLSM, showing that DAS@CD‐OxPt (IV) NPs + anti‐PD‐1 antibody group induced much more CD4^+^ and CD8^+^ T cells infiltration compared with the control group (Figure 6i,j; Figure S28, Supporting Information). In addition to DCs and T cells, macrophages (TAMs) play an important role in antigen presentation and engulfing cancer cells. The collected data showed that compared with the control group, the DAS@CD‐OxPt (IV) NPs + anti‐PD‐1 antibody group significantly reduced the production of Tregs to 18.44% (0.17 times) in the tumors (Figure S29, Supporting Information). Analysis of flow cytometry showed that the ratio of M1/M2 macrophages of DAS@CD‐OxPt (IV) NPs (0.73) and DAS@CD‐OxPt (IV) NPs + anti‐PD‐1 antibody (1.20) were 2.53 and 4.13 times than that of the control group, indicating that M2 macrophages into M1 macrophages have the potential to enhance the immune response (Figure S30 and S31, Supporting Information).

Moreover, enzyme‐linked immunosorbent assay (ELISA) was used to detect the tumor immune‐related factors, including tumor necrosis factor‐α (TNF‐α) and interferon‐γ (IFN‐γ), to evaluate the chemotherapy‐mediated immune response in vivo. Increased observable expression of TNF‐α and IFN‐γ were detected in the blood of mice treated with DAS@CD‐OxPt (IV) NPs + anti‐PD‐1 antibody, which was more effective than other treatments (Figure S32, Supporting Information). These results manifested that the chemo‐immunotherapy by integrating DAS@CD‐OxPt (IV) NPs with anti‐PD‐1 antibody was highly desirable for tumor suppression.

Encouraged by the effective combination therapy and antitumor immune response of the DAS@CD‐OxPt (IV) NPs + anti‐PD‐1 antibody, we investigated whether combined strategies can enhance tumor suppression and restrain metastasis in lung metastasis models by the continuous immune response. Definitely, the DAS@CD‐OxPt (IV) NPs + anti‐PD‐1 antibody treatment group was still the best treatment group among all treatment groups, and there was no significant difference in the body weight of mice in each group (Figures S33 and S34, Supporting Information). The bright field pictures and H&E sections of the lungs, lung metastatic nodules, and lung weight showed that the mice of control and anti‐PD‐1 antibody groups showed serious pulmonary metastasis, and the proportion of pulmonary metastatic nodules were 60.09% and 42.33%, respectively (Figure 6l,m). Furthermore, the proportion of pulmonary metastatic nodules in the control group was 3.25 and 7.54 times that DAS@CD‐OxPt (IV) NPs and DAS@CD‐OxPt (IV) NPs + anti‐PD‐1 antibody groups, DAS@CD‐OxPt (IV) NPs + anti‐PD‐1 antibody group shown a terrific talent for adaptive and standing immune responses (Figure 6n). Taken together, DAS@CD‐OxPt (IV) NPs had a robust systemic antitumor immune effect by promoting DCs maturation and T cell infiltration, and synergized with anti‐PD‐1 antibody to strikingly suppress CT26 tumor growth and lung metastasis, achieving potent inhibition of tumor progression, eventually.

After incubation with the high concentrations of DAS@CD‐OxPt (IV) NPs (200 µM) at 37 °C, the hemolysis rates of mouse erythrocytes were all lower than the threshold value of 5%, suggesting that it could normally participate in blood circulation, which laying a good foundation for biological experiments (Figure S35, Supporting Information). According to the results of the blood routine analysis, most of the blood routine parameters remained within normal ranges following the administration of OxPt (II), DAS, Mix, and DAS@CD‐OxPt (IV) NPs. However, certain parameters in Mix were found to be outside the normal range, including WBC, lymph, RBC, HGB, and HCT. This could potentially be attributed to a lack of carrier or side effects induced by these drugs on the biological organism. Meanwhile, the relevant blood biochemical indexes were simultaneously assessed, and the test results conformed to the established standards for mice without any evident abnormalities (Figure S36, Supporting Information).

## 3 Conclusion

In this work, we report a dual stimulus‐responsive supramolecular nanocomplex strategy that provides an efficient sequential infiltration driven by neighboring effect enabling deep tumor penetration and synergetic chemo‐immunotherapy for colorectal cancer. The expected DAS@CD‐OxPt (IV) NPs are able to regulate the release activation and expected sites of chemotherapeutics drugs in response to low acidity and high GSH in TME. The unchained OxPt (II) and DAS together stabilize the expression of p53 in cells, arrest the cell cycle, induce multiple damage to DNA, and enhance the neighboring effect to achieve deep penetration and invasion of drugs into tumors. Importantly, this supramolecular nanocomplex‐mediated chemotherapy elicits a remarkable ICD effect to facilitate systemic antitumor immunity. Specifically combined with anti‐PD‐1 antibody, it can efficiently inhibit primary tumor growth, distant tumor growth, pulmonary metastasis of CRC, and markedly prolong survival of CT26 tumor‐bearing mice attributed to activation and infiltration of various immune cells. The present supramolecular assembly strategy can be excavated a significant mission by facing different drugs combination and other cancer types, leading a fascinating prospect when nanomedicine meets cancer.

## 4 Experimental Section

### Ethics Statement

Animal experiments were performed according to the protocol approved by the Institutional Animal Care and the Southwest University Use Committee (IACUC) (license no. IACUC‐20231215‐03).

### Materials

Mono‐(6‐amino‐6‐deoxy)‐β‐cyclodextrin (β‐CD‐NH_2_) was purchased from Ruixi Biotechnology Co., Ltd (Xi'an, China). Oxaliplatin (OxPt), Dasatinib (DAS), 4‐dimethylaminopyridine (DMAP), N, N‐Dimethylformamide (Anhydrous, DMF), 1‐(3‐Dimethylaminopropyl)−3‐ethylcarbodiimidehydrochloride (EDC), Succinic anhydride (SA) and 3‐(4,5‐dimethylthiazol‐2‐yl)−2,5‐diphenyltetrazolium bromide (MTT) were supplied by Sigma‐Aldrich (USA). Hydrogen peroxide 30% (H_2_O_2_), Acetic acid, and Dichloromethane were Adamas Reagent Co., Ltd., Lyso Tracker Red, HMGB1 Assay Kit, CRT Assay Kit, γ‐H2AX and DAPI were all supplied by Beyotime Biological Co., Ltd. (Shanghai, China). All other chemicals and reagents used were of analytic grade. In vivo, images of fluorescence imaging and distribution were recorded by Living Animal and Plant Imaging System (IVIS Lumina Series III, PERKINELMER, USA).

### Synthesis of Monocarboxylated Oxaliplatin (OxPt‐COOH)

The mono‐carboxylated oxaliplatin was synthesized according to a previous report from Gregory Thiabaud et al. ^[^ ^49^ ^]^ OxPt (II) (500 mg, 1.25 mmol) was dispersed in 10 mL acetic acid, 0.5 mL hydrogen peroxide (H_2_O_2_, 30%) was added in the mixture. The above suspension was stirred away from light at room temperature (RT) for 24 h to obtain a colorless transparent solution. After vacuum drying at 45 °C, the solvent was removed and white solid powder (OxPt‐Ac (IV)) was obtained. In the Ar atmosphere, OxPt‐Ac (IV) (470 mg, 1 mmol) was dispersed in 8 mL of N, N‐dimethylformamide (DMF), then 2 mL of DMF dissolved with succinic anhydride (200 mg, 2 mmol) was added drop by drop, heated to 50 °C, and stirred overnight. The tan solution was vacuum‐dried overnight and then suspended in 10 mL of acetone. The suspension was filtered, the filtrate was dried under vacuum, the crude product was purified by silica gel column chromatography, and the final product was obtained by gradient rinsing of methylene chloride, methylene chloride: methanol (10:1, v/v), methylene chloride: methanol (5:1, v/v) and methylene chloride: methanol (2:1, v/v). (Yield: 47%)

### Synthesis of Cyclodextrin Prodrug (CD‐OxPt (IV))

Under the protection of Ar, OxPt‐COOH (114 mg, 0.2 mmol), DMAP (35 mg, 0.3 mmol) and EDC (57.6 mg, 0.3 mmol) were dissolved in 5 mL DMF, and then 2 mL of DMF dissolved with mono‐(6‐amino‐6‐deoxy)‐β‐cyclodextrin (β‐CD‐NH_2_) was added in an ice bath and stirred at the RT for 48 h. At the end of the reaction, the coarse product was precipitated in excess ether many times to obtain the brown product. (Yield: 83%)

### Preparation of DAS@CD‐OxPt (IV) NPs

CD‐OxPt (IV) (39.51 mg, 23.4 µmol) and DAS (11.42 mg, 23.4 µmol) were dispersed in 5 mL 70% ethanol, condensed at 75 °C for reflux until the solids were completely dissolved, and stirred continuously for 24 h to make host and guest complex. After DAS@CD‐OxPt (IV) NPs were formed, the solution was cooled slowly to room temperature. The reaction temperature and speed were carefully controlled to prevent DAS@CD‐OxPt (IV) NPs from flocculating again. The DAS@CD‐OxPt (IV) NPs is collected after removal of larger particle through centrifugation at low speed.

### Characterization of DAS@CD‐OxPt (IV) NPs

The NMR spectra including ^1^H NMR, and NOESY NMR were recorded on the 600 MHz NMR (BRUKER, Switzerland). X‐ray diffraction patterns for products were separately measured on the XRD‐7000 (SHIMADZU, China). The high‐resolution mass spectrum of synthetic products was chronicled by the impact II mass spectrometers (BRUKER, Germany). XPS spectra were recorded on the X‐ray photoelectron spectroscopy (Thermo Scientific K‐Alpha, USA). The DSC analysis was conducted on a STA409PC differential thermal analyzer (NETZSCH, Germany). The heating was carried out at the rate of 10 °C min^−1^ under a N_2_, and the scanning ranged from 50 to 350 °C. The TG analysis was recorded on a TGA‐Q50 thermal gravimetric analyzer (Waters, USA). FT‐IR spectra were recorded by the Thermo Nicolet 6700 FT‐IR spectrophotometer (Thermofisher, USA). The morphology of DAS@CD‐OxPt (IV) NPs in PBS 7.4 or 5.0 was observed by the JEM‐1230EX TEM (JEOL, Japan). The hydrodynamic diameter and polydispersity index (PDI) were measured by a Zetasizer Nano ZS90 dynamic light scattering (DLS) measurements (Malvern Instruments, UK). The UV‐vis spectra were derived from the UV‐1800 spectrophotometer (Shimadzu, Japan). The fluorescence spectra were recorded by FluoroMax+ fluorescence spectrometers (HORIBA, USA).

### Drug Release from the DAS@CD‐OxPt (IV) NPs

DAS@CD‐OxPt (IV) NPs with the concentration of 200 µg/mL was dispersed in PBS. The 2 mL solution was sealed in the dialysis tube with the molecular weight cut‐off of 3.5 kDa and dialyzed against PBS in the presence of pH 7.4 or 5.0 for 24 h. The 1 mL of the solution was taken out at designated time intervals and the equal volume of fresh media was added. The released amounts of DAS were quantified according to the UV‐vis spectrometer as aforementioned.

### Study on the Dissociation Fluorescence Responses of DAS@CD‐OxPt (IV) NPs

The GSH response of Nile Red labeled DAS@CD‐OxPt (IV) NPs was measured fluorescence. A mixed solution containing known amounts of Nile Red@NPs (0.3 mg mL^−1^) was added in PBS 7.4 with 10 mM GSH at different time points. The fluorescence curves were measured by the fluorescence spectrometers as aforementioned. The fitting of data from dissociation fluorescence decay curves was performed in a nonlinear manner and the dissociation half‐life period was obtained by fitted curve.

### Rigid Scanning and Quantum Chemical Computation

According to the experimental results of the synthesis of β‐cyclodextrin derivatives (CD‐OxPt (IV)), the geometric configuration of CD‐OxPt (IV) was constructed by GaussView 5.0.9. The accurate structure of DAS (ID: 3062316) was acquired from the PubChem database (https://pubchem.ncbi.nlm.nih.gov/). The Gaussian 09 software was used to select the theoretical method and base group B3LYP‐D3(BJ)/def2svp for the geometric optimization and vibration analysis of the initial configuration of the above CD‐OXA and DAS molecules in the ethanol environment, so as to obtain the structure without virtual frequency.

### Rigid Scanning

The circle surrounded by oxyglycoside atoms of CD‐OxPt (IV) was set onto the XY plane, and its center was defined as the center of the coordination system. The vertical direction of the wide circle is set as the Z‐axis. The optimized ligand molecules move 6Å distance to the center of the coordination system along the Z axis at a step size of 0.15 Å per step. In the process of moving, the B3LYP‐D3(BJ)/def2svp method was devoted to calculating the single‐point energy of the structure generated in each step, and the relative energy (Erel) was obtained by subtracting the single‐point energy from the initial energy. After the stable conformation was obtained, the CPCM ethanol solvent model was selected while the ORCA 5.0.3 program^[^ ^50^ ^]^ was used to calculate the high precision single point energy at the RI‐B3LYP‐D3(BJ)/def2‐TZVP level to obtain the free energy of the DAS@CD‐OxPt (IV).

### Binding Energies and EDA‐FF

On the basis of obtaining the stable conformation of the DAS@CD‐OxPt (IV), the single point energy was calculated at the theoretical level of RI‐B3LYP‐D3(BJ)/def2‐TZVP, and the binding energy of the complex was calculated using the following equation.

(1) $$E_{\mathrm{binding}}=E_{\mathrm{DAS}@\mathrm{CD}-\mathrm{OxPt}\;(\mathrm{IV})}\;\left(E_{\mathrm{CD}-\mathrm{OxPt}\;\left(\mathrm{IV}\right)}+E_{\mathrm{DAS}}\right)$$

where E_DAS@CD‐OxPt (IV)_, E_CD‐OxPt (IV),_ and E_DAS_ represent the free energy of DAS@CD‐OxPt (IV), CD‐OxPt (IV), and DAS in ethanol solvent, respectively.

In order to quantitatively analyze the weak interactions between DAS@CD‐OxPt (IV) molecules, refer to Lu et al., Multiwfn 3.8 (dev)^[^ ^51^, ^52^ ^]^ software was used to carry on the energy decomposition analysis based on force field (EDA‐FF) analysis.

### Interaction Region Indicator (IRI)

In order to visually display the region of interaction between CD‐OxPt (IV) and DAS molecules in the DAS@CD‐OxPt (IV), IRI analysis was performed on the wave function generated by the stable structure of the DAS@CD‐OxPt (IV) at the level of RI‐B3LYP‐D3(BJ)/def2‐TZVP by reference to Lu et al. The VMD (version: 1.9.3) program (Humphrey, Dalke, & Schulten, 1996) visualizes the results of the analysis.

### Atoms in Molecules (AIM)

To obtain information on the strength of complex interactions and intermolecular hydrogen bonds, the Multiwfn 3.8(dev) and VMD (version: 1.9.3) program carried out topological analysis of AIM theory (Bader, 1985) on the generated wave function calculated at RI‐B3LYP‐D3(BJ)/def2‐TZVP theoretical level of theory.

### Cytotoxicity Evaluation of DAS@CD‐OxPt (IV) NPs

The cytotoxicities of DAS, OxPt, Mix, DAS@CD‐OxPt (IV) NPs (0, 5, 10, 20, 30, 40 µM) against tumor cells were assessed by MTT assay. The absorbance of treatment groups at 490 nm was measured with a microplate reader (SPARK‐10 M, Switzerland) to determine the cell viability.

### Colony Formation Assay of DAS@CD‐OxPt (IV) NPs

CT26 cells were incubated in 6‐well plates overnight with 2 × 10^3^ cells per well. Then, the cells were incubated with serum‐free DMEM, DAS, OxPt, Mix, DAS@CD‐OxPt (IV) NPs (5 µM) for 12 h. Subsequently, the cells were persistently incubated in the presence of fresh culture medium replacing various drugs for 2 weeks. The clones were fixed with tissue fixing solution for 30 min and stained with crystal violet staining solution for 20 min. The relative clone proportion (%) was obtained using calculating the ratio of the crystal violet staining areas of the control and experimental groups.

### Detection of Cell Cycle Arrest and Cell Apoptosis

CT26 cells were seeded in 12‐well plates (1.5 × 10^5^ cells/well) overnight. Cell cycle and cell apoptosis assay were detected by cell cycle and apoptosis analysis kit (Beyotime Biotechnology). Then, the cells were treated with PBS, DAS, OxPt, Mix, DAS@CD‐OxPt (IV) NPs (20 µM) for 24 h. In cell cycle arrest assay, the cells were collected, washed with PBS, and fixed with 75% ethanol for another 12 h. Subsequently, the cellular dye was added to the cells for 0.5 h and recorded by flow cytometry (NovoCyte 2060R, USA).

### Live/Dead Assay in 2D/3D Multicellular Spheroids (MCSs)

CT26 tumor multicellular spheroids were incubated according to previously reported.^[^ ^53^ ^]^ The 2D/3D spheroids were treated with PBS, DAS, OxPt, Mix, DAS@CD‐OxPt (IV) NPs (20 µM) for 24 h. Subsequently, the MCSs were washed with PBS, and the spheroids were stained with FDA/PI. Subsequently, the fluorescence of live/dead images was evaluated with CLSM.

### Assay of Neighboring Effect

The cellular uptake of FITC@NPs in CT26 cells estimating the neighboring effect of NPs. CT26 cells were seeded in 96‐well plates (1 × 10^4^ cells/well) overnight. The cells were treated FITC@NPs (10 µM) for 12 h, and then the supernatant was collected. The supernatant and the same concentration of FITC@NPs as the supernatant were added into the new 96‐well plates for 10 min, 2 h, 4 h, then the fluorescence of the cells was determined with FCM.

The MTT assay was employed to evaluate the neighboring effect of NPs. CT26 cells were incubated in 6‐well plates overnight with 1 × 10^4^ cells per well. The cells were treated DAS@CD‐OxPt (IV) NPs (20 µM) for 12 h, and then the collected supernatant and DAS@CD‐OxPt (IV) NPs (same concentration as the supernatant) were added to the new 96‐well plates for 2, 6, 12 h. The optical absorbance was determined with a microplate reader at 490 nm.

The penetration of FITC@NPs in CT26 MCSs evaluating the neighboring effect of NPs. CT26 cells were seeded in 96‐well plates (1 × 10^4^ cells/well) overnight. The FITC@NPs co‐incubates with cells and untreated FITC@NPs were added into the formed tumor spheroids, and the permeability was observed by CLSM.

### Analysis of DNA Damage and p53 Expression

CT26 cells were seeded in 24‐well plates (5 × 10^4^ cells/well) overnight. The cells were treated with PBS, DAS, OxPt, Mix, DAS@CD‐OxPt (IV) NPs (20 µM) for 24 h, washed twice with PBS, and fixed 20 min with tissue fixing solution, penetrated 10 min with 1% Triton X‐100, blocked 2 h with 5% BSA, and then incubated with γ‐H2AX or p53 primary antibody at 4 °C for 24 h, respectively. After washing twice with PBS, cells were incubated with Alexa Fluor 488 or 647‐conjugated antibody for 1 h. Cell nuclei were stained with DAPI and imaged using CLSM.

### Immunofluorescence Staining

Immunofluorescence staining of CT26 cells was carried out as previously reported,^[^ ^54^ ^]^ and then incubated with CRT or HMGB1 primary antibody at 4 °C for 24 h, respectively. After washing twice with PBS, cells were incubated with Alexa Fluor 488 or 647‐conjugated antibody for 1 h. Cell nuclei were stained with DAPI and imaged using CLSM.

### In Vitro Determination of DC Maturation

Murine bone marrow‐derived dendritic cells (BMDCs) were obtained by the femur and tibia of 6‐week‐old female BALB/C mice. BMDCs cultured with a half‐changed solution for 5 days in RPMI 1640 medium supplement with 10% FBS, GM‐CSF (20 ng mL^−1^, Beyotime Biotechnology), and IL‐4 (10 ng mL^−1^, Beyotime Biotechnology). After that, the cells in suspension were collected and added to the lower layer of a 24‐well Transwell culture plate with a density of 1 × 10^6^ cells/well. CT26 cells with different drugs treated were incubated in a 24‐well Transwell culture plate's upper layer cultured for 24 h, and the DCs of the lower layer were collected, blocked 1 h with 5% BSA, and the cells were labeled by the following antibodies: anti‐mouse CD11c‐PE‐Cy7, anti‐mouse CD80‐APC, anti‐mouse CD86‐PE (BioLegend). Finally, cells were washed with PBS and detected by FCM.

### In Vivo Fluorescence Imaging and Distribution

Briefly, 100 µL of cells (2 × 10^6^) was injected into the subcutaneous dorsal of BALB/C mice to establish a solid CT26 cancer‐bearing mice model. The mice were intravenously injected with DiR, DiR‐labeled DAS@CD‐OxPt (IV) NPs (Dir@NPs, Dir: 10 mg kg^−1^) when the tumor volume grew ≈150 mm^3^. The Dir fluorescence signal was captured by IVIS Lumina Series III Plant living imaging system (PerkinElmer, USA) at presupposed time points (2, 6, 12, 24, and 48 h). Then, the mice were sacrificed and dissected, and the tumors and major organs were collected and subjected to fluorescence imaging. In addition, tumor sections were subjected to DAPI staining and evaluated penetration of DAS@CD‐OxPt (IV) NPs in tumors by CLSM.

### In Vivo Antitumor Therapeutic Potential Evaluation

The CT26 tumor‐bearing mice were optionally divided into control, DAS, OxPt, Mix, DAS@CD‐OxPt (IV) NPs (DAS: 5 mg kg^−1^, OxPt: 5 mg kg^−1^) five groups consisting of five mice in each group when the tumor volume approximately 100 mm^3^. Subsequently, tumor volume was recorded by a vernier caliper measured by employing the following equation: tumor volume = (tumor length) × (tumor width)^2^ × 0.5. On day 14, the mice were sacrificed and dissected, the tumor tissues and major organs were removed and weighted, and histopathological analysis of major tumor tissues was performed by H&E, TUNEL, Ki67, CD31, and immunofluorescence staining.

### In Vivo Combination Antitumor Efficacy with anti‐PD‐1 Antibody and Survival Studies

Based on the previous studies,^[^ ^55^ ^]^ the combination antitumor efficacy assay with anti‐PD‐1 antibody was determined survival. After 15 days, the CT26 cells (2 × 10^6^ cells) surviving BALB/c mice (*n* = 5) after combination anti‐PD‐1 antibody treatment were injected subcutaneously with 5 × 10^5^ CT26 cells (left flank) as distant tumors on day 15. In a humanitarian setting, mice were euthanized when any of the following conditions occurred to the BALB/c mice: 1) weight loss or gain of more than 20%, 2) moribund, 3) severe abdominal distension and inability to move, or 4) tumor volume exceeded 1800 mm^3^.

### In Vivo Immune Activation

To verify that anti‐PD‐1 antibody treatment strategy significantly enhanced immune activation, CT26 tumor‐bearing mice received various treatments including control (G1); anti‐PD‐1 antibody (G2); DAS@CD‐OxPt (IV) NPs (G3); DAS@CD‐OxPt (IV) NPs + anti‐PD‐1 antibody (G4) (DAS: 5 mg kg^−1^, anti‐PD‐1 antibody: 10 mg kg^−1^) every three days and the treatment was repeated 4 times. After the end of treatment, the tumors and lymph nodes were harvested. The tissues were processed as single‐cell suspensions, and single‐cell suspension was labeled by the following antibodies: anti‐mouse CD11c‐PE‐Cy7, anti‐mouse CD80‐APC, and anti‐mouse CD86‐PE. For the analysis of T cells, the single‐cell suspension was labeled by the following antibodies: anti‐mouse CD3‐APC, anti‐mouse CD4‐FITC, and anti‐mouse CD8‐PE. For the analysis of TAMs cells, the single‐cell suspension was labeled by the following antibodies: anti‐mouse F4/80‐APC, anti‐mouse CD11b‐FITC, anti‐mouse CD206‐PE‐Cy7, anti‐mouse CD80‐PE. For the analysis of Treg cells, the single‐cell suspension was labeled by the following antibodies: anti‐mouse CD4‐PE‐Cy7, anti‐mouse CD3‐FITC, and anti‐mouse Foxp3‐PE. Finally, the infiltrating immune cells were detected by FCM.

### In Vivo Antimetastasis Evaluation

The anti‐metastasis evaluation of combination therapy was observed on the pulmonary metastasis model of CT26‐tumor‐bearing BALB/c mice. After 15 days post‐treatment, the pulmonary metastasis model was established by injecting CT26 cells (3 × 10^5^ CT26 cells per mouse) with i.v. injection. Then, the 15 days of the injection of cells, the lungs were collected and fixed with tissue fixative for further H&E staining, the lung images were recorded and the pulmonary metastasis nodules were calculated.

### Hemolysis Analysis

Hemolysis analysis is performed according to previous experimental procedures.^[^ ^56^ ^]^ The absorbance curves of supernatant including broken hemoglobin were measured by UV‐1800 spectrophotometer.

(2) $$\mathrm{Hemolysis}\;(\%)=\frac{\mathrm{OD}\;\mathrm{treatment}\;\mathrm{group}-\mathrm{OD}\;\mathrm{negative}\;\mathrm{group}}{\mathrm{OD}\;\mathrm{positive}\;\mathrm{group}-\mathrm{OD}\;\mathrm{negative}\;\mathrm{group}}\times 100\%$$

where OD treatment group, OD negative group, and OD positive group were the absorbance values at 576 nm of the test sample, negative control (PBS), and positive control (1% Triton X‐100), respectively.

### Statistical Analysis

Data are given as mean ± standard deviation (SD). Flow cytometry results were analyzed with NovoExpress. The fluorescence images were analyzed with ZEISS‐ZEN or Living Image software. All statistical analyses were performed using GraphPad Prism 8.0 software and statistical significance was calculated via two‐way ANOVA or one‐way ANOVA. *p* < 0.05 was considered statistically significant, *p < 0.05, **p < 0.01, ***p < 0.001.

## Conflict of Interest

The authors declare no conflict of interest.

## Author Contributions

Z. X., M.Y., and J.H. conceived and designed the experiments (with equal contributions). The majority of the experiments were assisted by L.H. and H.Z. The data analysis and manuscript were prepared by M.Y., J.H., P. X., S.B., Y. K., and Z. X. All authors have discussed the results and approved the final manuscript.

## Supporting information

Supporting Information

## Data Availability

The data that support the findings of this study are available from the corresponding author upon reasonable request.
