# Supplementary material for: Neighboring Effect‐Initiated Supramolecular Nanocomplex with Sequential Infiltration as Irreversible Apoptosis Inducer for Synergetic Chemo‐Immunotherapy
Source: Adv Sci (Weinh). 2024 Aug 13;11(38):2402809. doi: 10.1002/advs.202402809 (PMC11481388; doi:10.1002/advs.202402809)
Supplement: Supplementary file 1 — Supporting Information [file ADVS-11-2402809-s001.docx]

Supporting Information

**Neighboring Effect-initiated** **Supramolecular Nanocomplex with Sequential Infiltration as Irreversible Apoptosis Inducer for Synergetic Chemo-immunotherapy**

Mengjie Ye^a^, Junfeng Hu^a^, Linlin Han^a^, Hengbo Zhang^a^, Peng Xue^a^, Yuejun Kang^a^, Shuang Bai*^b^, and Zhigang Xu*^a,c,d,e^

^a^Key Laboratory of Luminescence Analysis and Molecular Sensing, Ministry of Education, School of Materials and Energy & Chongqing Engineering Research Center for Micro-Nano Biomedical Materials and Devices, Southwest University, Chongqing 400715, P. R. China.

^b^State Key Laboratory of Chemo/Biosensing and Chemometrics, Hunan University, Changsha 410082, P. R. China.

^c^Shaanxi Province Center for Regenerative Medicine and Surgery Engineering Research, the First Affiliated Hospital of Xi’an Jiaotong University, Xi’an 710061, China.

^d^Yibin Academy of Southwest University, Yibin 644000, China.

^e^Key Laboratory of Laser Technology and Optoelectronic Functional Materials of Hainan Province College of Chemistry and Chemical Engineering, Hainan Normal University, Haikou 571158, China.

Email: Shuang Bai ([shuangbai@xjtufh.edu.cn](mailto:shuangbai@xjtufh.edu.cn)); Zhigang Xu (zgxu@swu.edu.cn)

# Supporting Figures


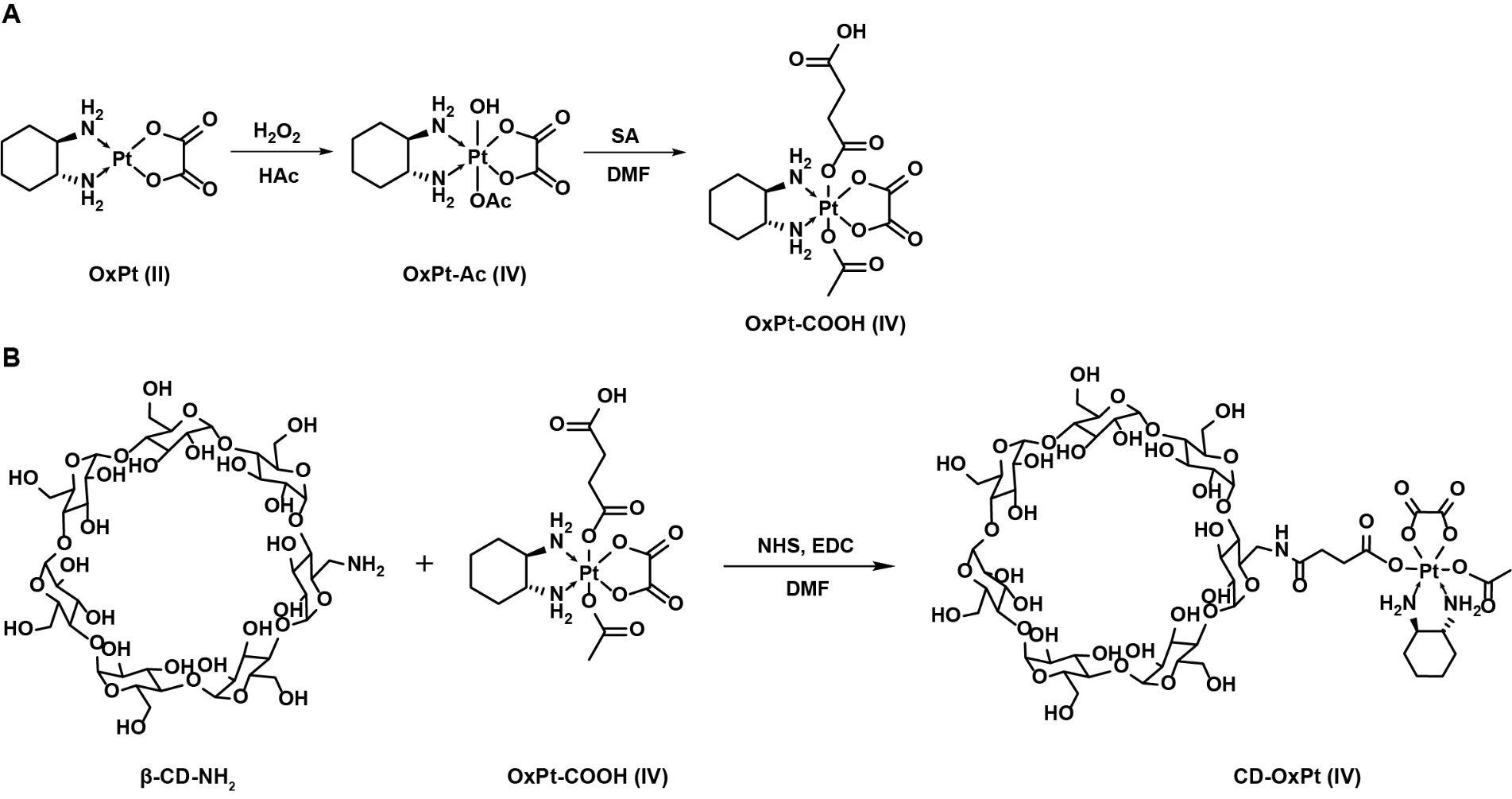


**Figure S1.** Synthesis and chemical structures of (A) OxPt-COOH (IV) and (B) CD-OxPt (IV).


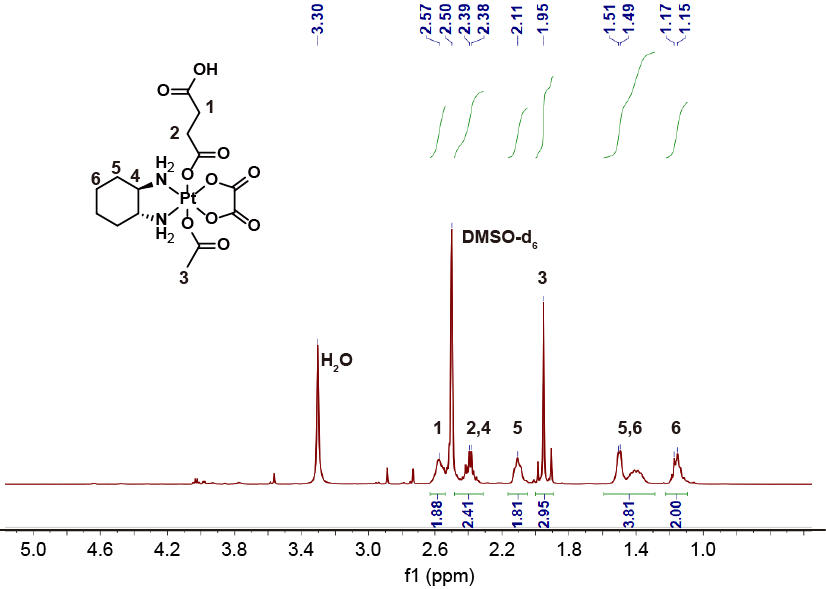


**Figure S2.** ^1^H NMR spectrum of OxPt-COOH (IV).

.


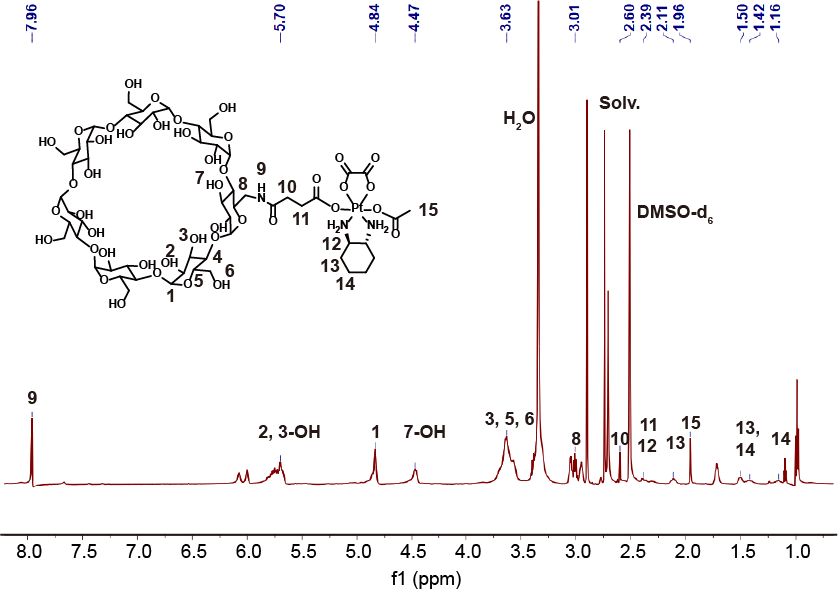


**Figure S3.** ^1^H NMR spectrum of CD-OxPt (IV).


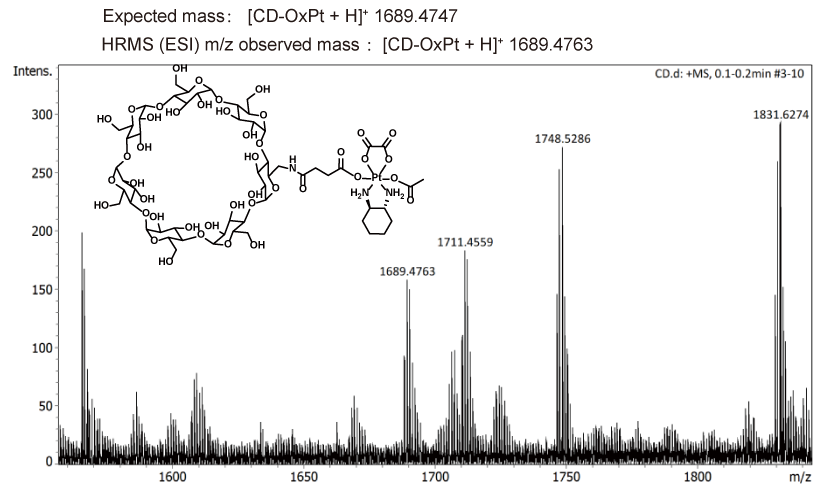


**Figure S4.** The high-resolution mass spectrum of CD-OxPt (IV).


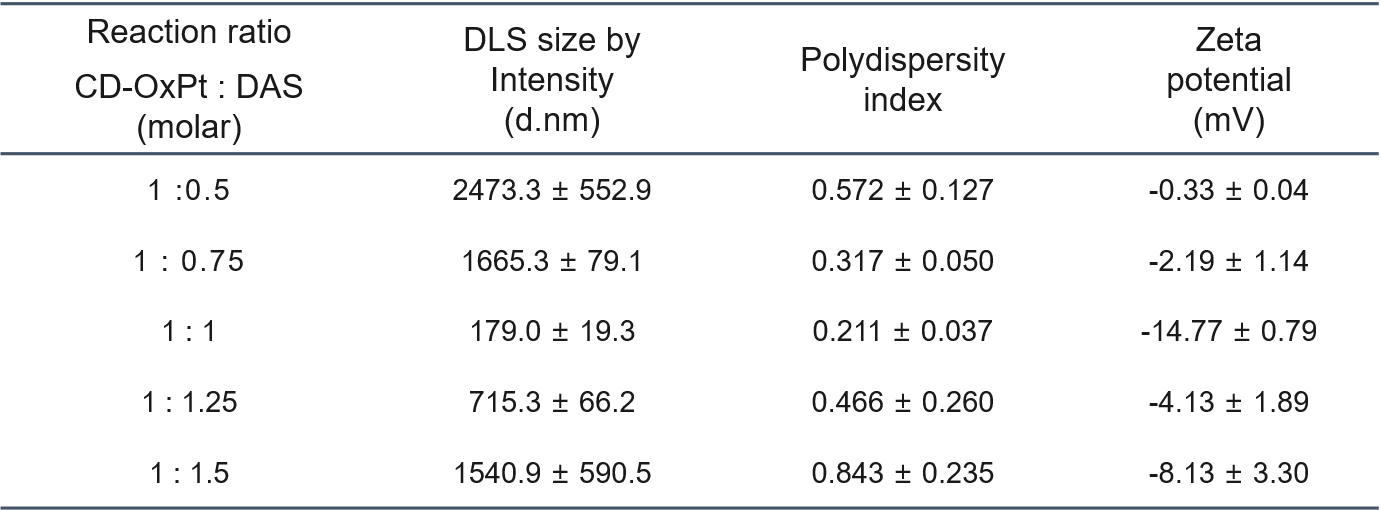


**Figure S5.** Hydrodynamic diameter, polydispersity index and zeta potential of DAS@CD-OxPt (IV) NPs under different molar ratios of DAS and CD-OxPt (IV) (n = 3).


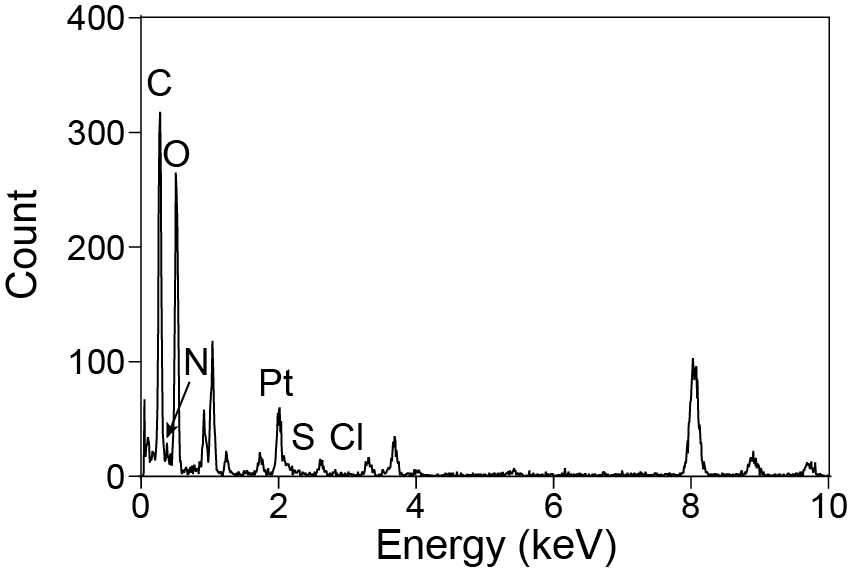


**Figure S6.** The proportion of elements in DAS@CD-OxPt (IV) NPs.


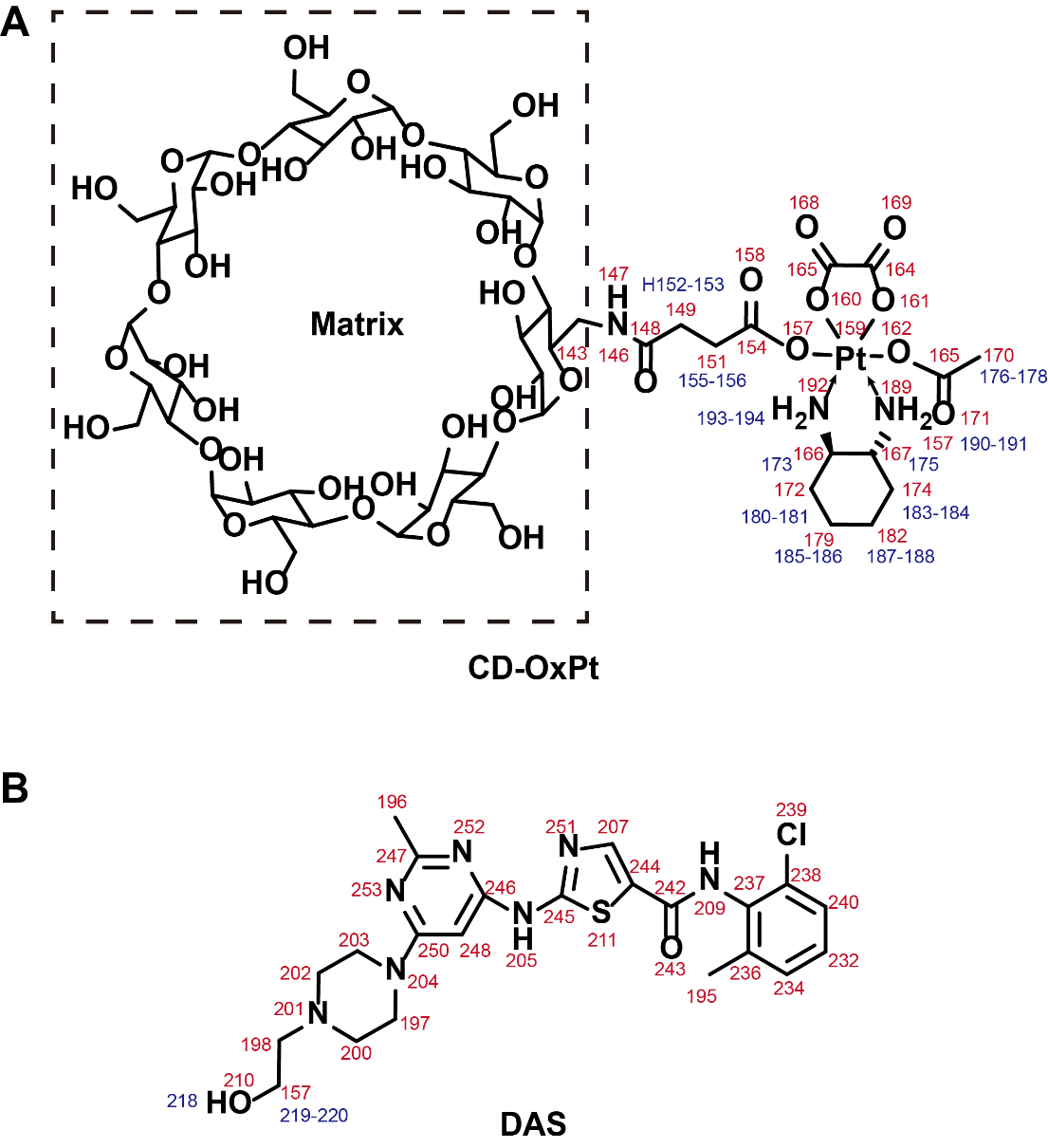


**Figure S7.** The associated atomic sequence number of CD-OxPt (IV) and DAS.

**Table S1.**

Table Topological parameters (in kJ/mol) for bonds of interacting atoms of DAS@CD-OxPt (IV) complexes

| BCP | Hydrogen bond | ρ(*r*) | ∇^2^ρ(*r*) | G(*r*) | V(*r*) | H(*r*) | E_HB_ |
| --- | --- | --- | --- | --- | --- | --- | --- |
| 294 | O243⋅⋅⋅H30 | 82.75 | 301.42 | 75.19 | −75.03 | 0.16 | −37.52 |
| 312 | O243⋅⋅⋅H28 | 32.30 | 137.07 | 28.63 | −23.00 | 5.63 | −11.50 |
| 330 | O243⋅⋅⋅H7 | 11.58 | 38.01 | 7.66 | −5.81 | 1.85 | −2.90 |
| 370 | O50⋅⋅⋅H212 | 17.33 | 66.52 | 13.16 | −9.69 | 3.47 | −4.85 |
| 391 | O3⋅⋅⋅H208 | 8.65 | 35.19 | 6.54 | −4.28 | 2.26 | −2.14 |
| 448 | N251⋅⋅⋅H133 | 35.44 | 105.70 | 22.62 | −18.80 | 3.81 | −9.40 |
| 511 | O71⋅⋅⋅H227 | 14.00 | 43.73 | 8.91 | −6.89 | 2.02 | −3.44 |
| 530 | O117⋅⋅⋅H206 | 34.23 | 131.86 | 26.44 | −19.91 | 6.53 | −9.96 |
| 543 | N205⋅⋅⋅H112 | 16.90 | 63.29 | 12.70 | −9.57 | 3.13 | −4.78 |
| 562 | O75⋅⋅⋅H230 | 25.09 | 88.33 | 18.18 | −14.28 | 3.90 | −7.14 |
| 577 | N252⋅⋅⋅H118 | 117.26 | 232.34 | 78.41 | −98.74 | −20.33 | −49.37 |
| 608 | O210⋅⋅⋅H76 | 115.23 | 308.82 | 95.40 | −113.60 | −18.20 | −56.80 |
| 661 | O92⋅⋅⋅H223 | 23.71 | 75.15 | 15.54 | −12.30 | 3.24 | −6.15 |
| 666 | O113⋅⋅⋅H216 | 23.66 | 89.95 | 18.28 | −14.08 | 4.21 | −7.04 |

BCP—bond critical point, ρ(*r*)—electron density, ∇^2^ρ(*r*)—Laplacian of electron density, G(*r*)—electron kinetic energy density, V(*r*)—electron potential energy density, H(*r*)—total electron energy density and E_HB_—hydrogen bond energy.


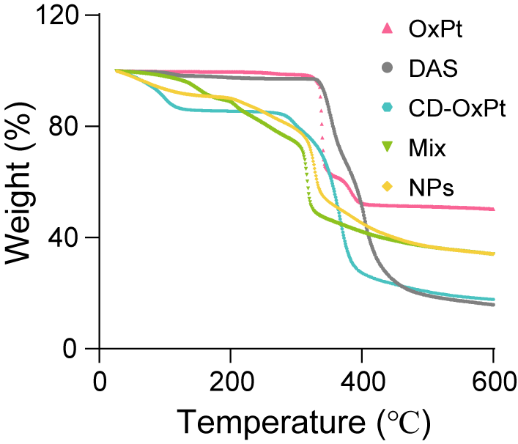


**Figure S8**. TGA curves of OxPt, DAS, CD-OxPt (IV), Mix, and DAS@CD-OxPt (IV) NPs.


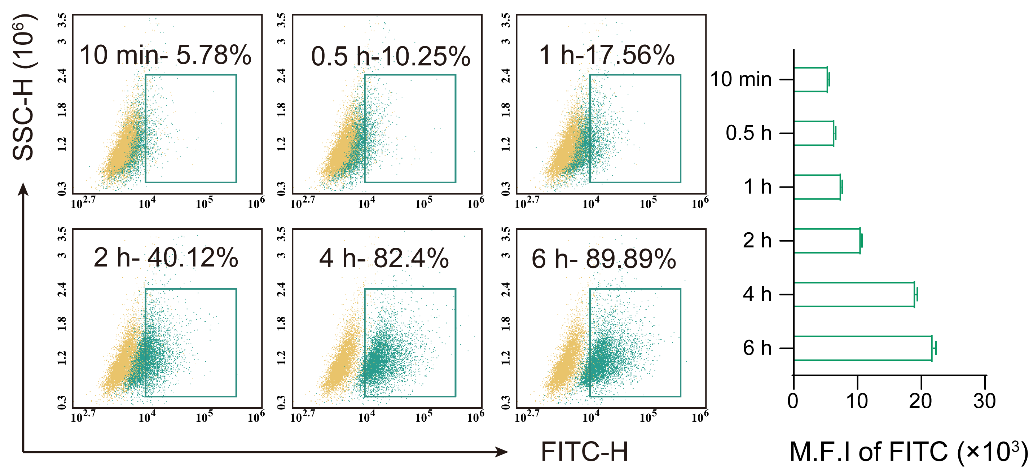


**Figure S9.** The cellular uptake and quantitative analysis of FITC@NPs measured at different time by flow cytometry (n = 3).


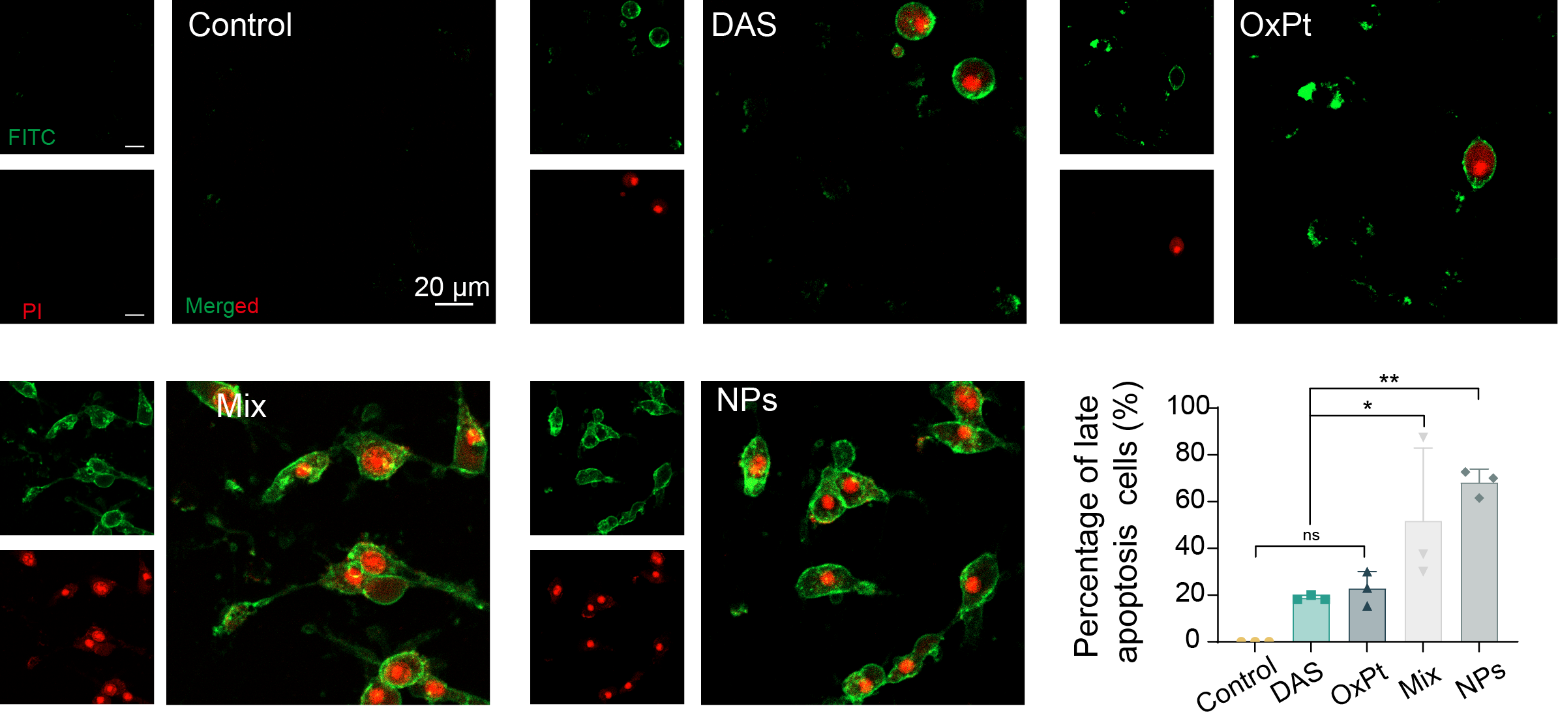


**Figure S10.** The CLSM images and quantitative analysis of CT26 cell apoptosis were treated with PBS, DAS, OxPt, Mix, DAS@CD-OxPt (IV) NPs (20 μM) for 24 h (n = 3). Scale bars = 20 µm.


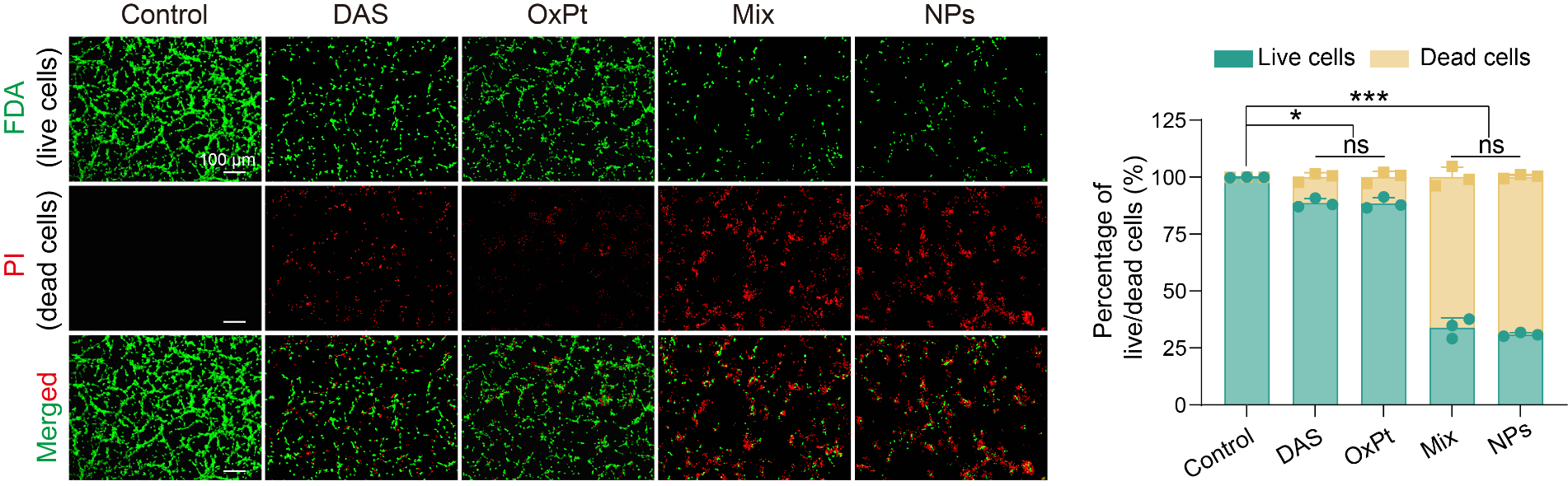


**Figure S11.** Live/Dead assay and quantitative analysis in CT26 cells treated with various drugs (20 μM) in vitro (n = 3). Scale bars = 100 µm.


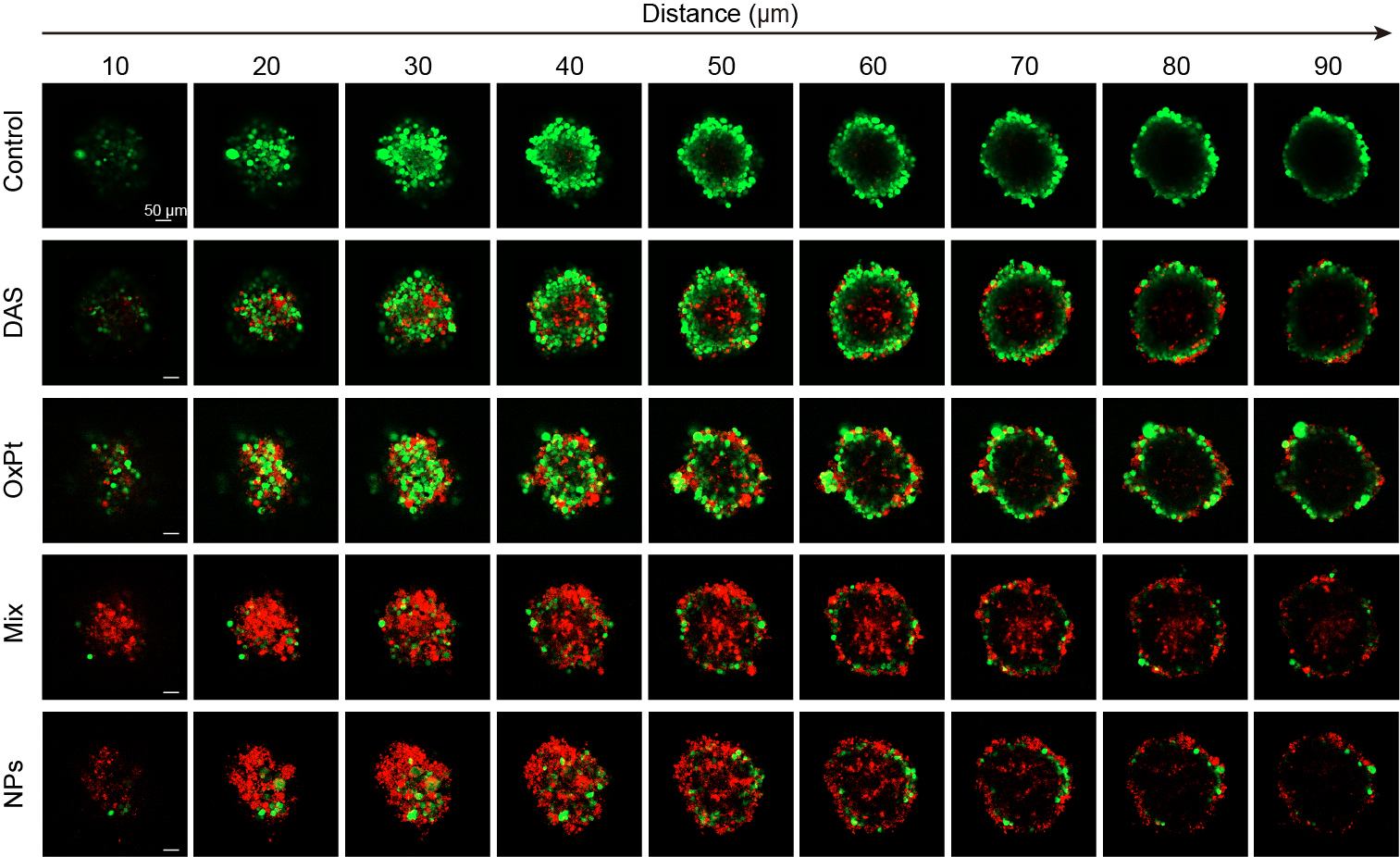


**Figure S12.** The images of live/dead assay at different penetration depth of CT26 MCSs after treatment with various formulations including PBS, DAS, OxPt, Mix, and DAS@CD-OxPt (IV) NPs (n = 3). Scale bars = 50 µm.


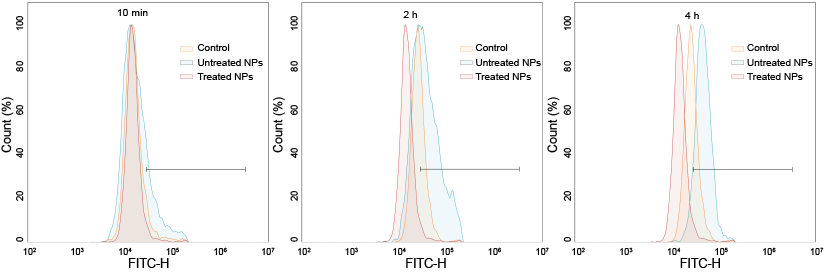


**Figure S13.** Cell uptake analysis of CT26 cells treated with co-incubated FITC@NPs (the spent medium from CT26 cells incubated with FITC@NPs for 12 h) or untreated FITC@NPs by flow cytometry (n = 3).


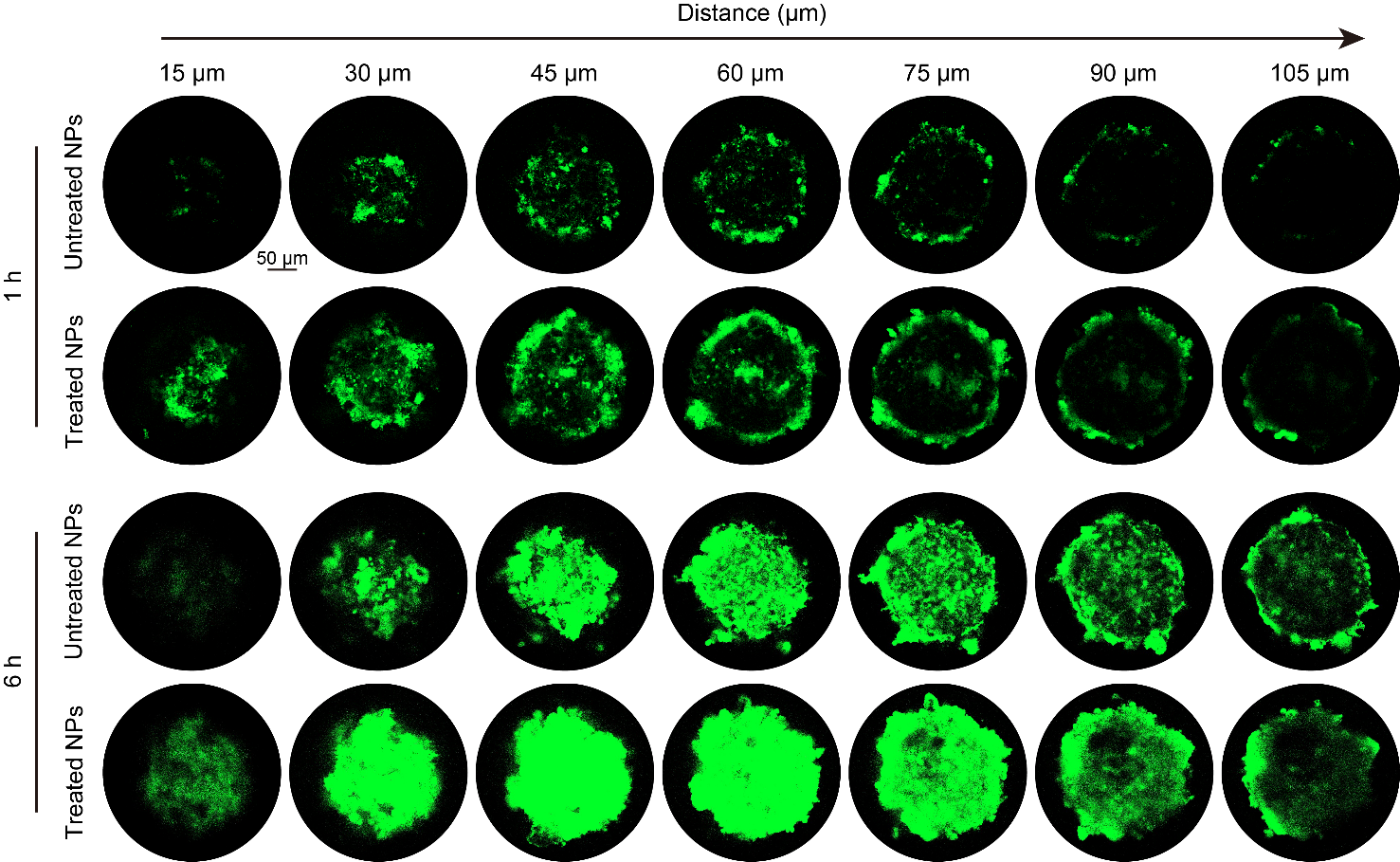


**Figure S14.** The penetrability at different penetration depths of CT26 MCSs after treatment with co-incubated FITC@NPs (the spent medium from CT26 cells incubated with FITC@NPs for 12 h) or untreated FITC@NPs (n = 3). Scale bar = 50 µm.


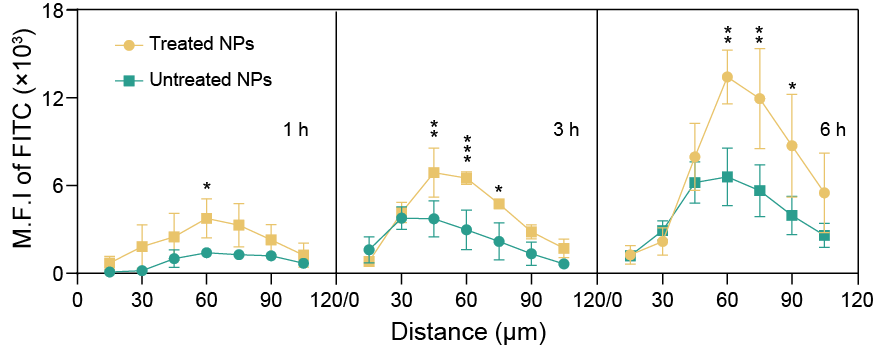


**Figure S15**. Quantification analysis of different penetration depths of CT26 MCSs after treatment with co-incubated FITC@NPs and untreated FITC@NPs (n = 3). Z-stack images using CLSM were obtained from the 15 μm to 105 μm of the tumor spheroid.


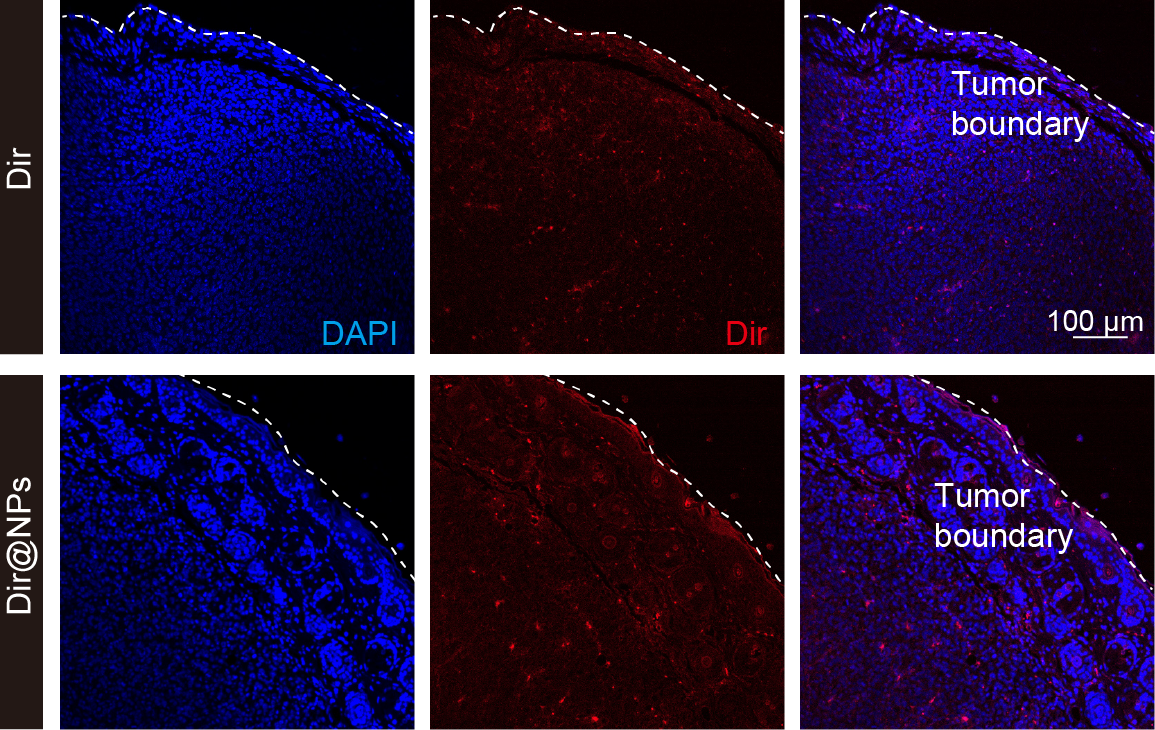


**Figure S16**. Fluorescence images of CT26 tumor tissues after injecting Dir and Dir@NPs at 12 h. Scale bar = 100 µm.


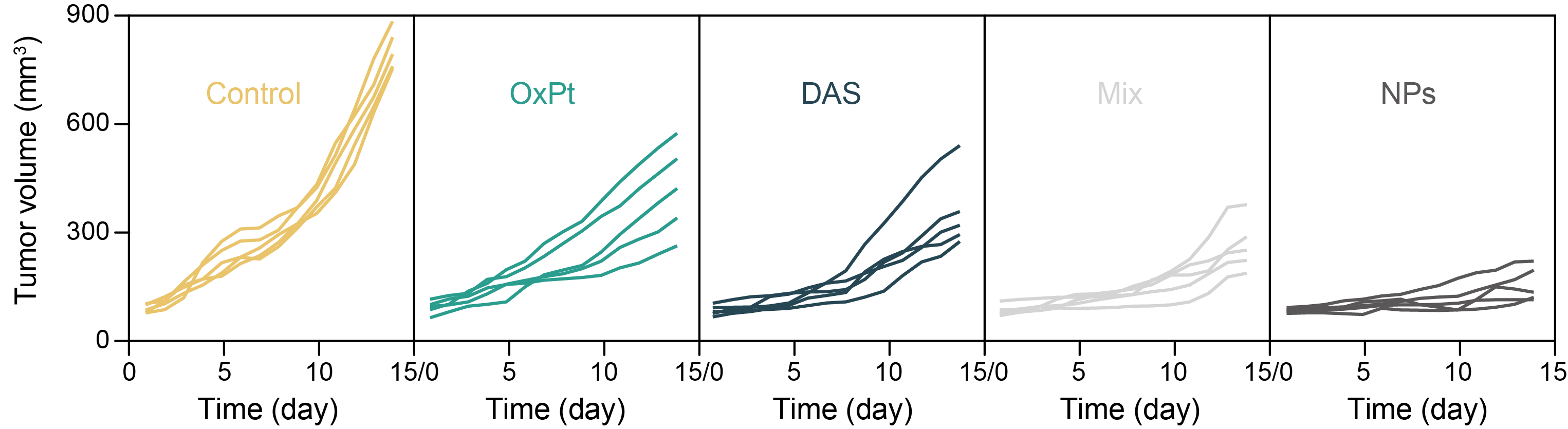


**Figure S17**. Individual CT26 tumor growth curves after different treatments (n = 5).


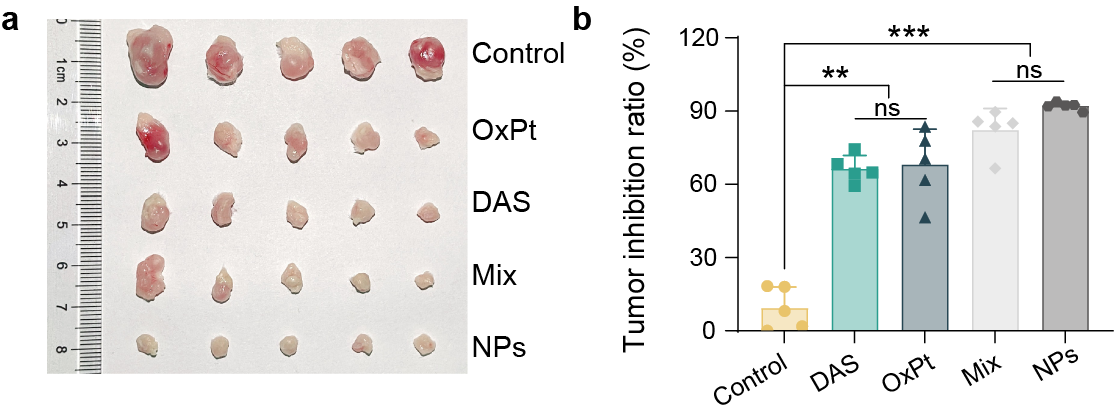


**Figure S18**. (a) Representative tumor photographs and (b) tumor inhibition ratio after different treatments (n = 5).


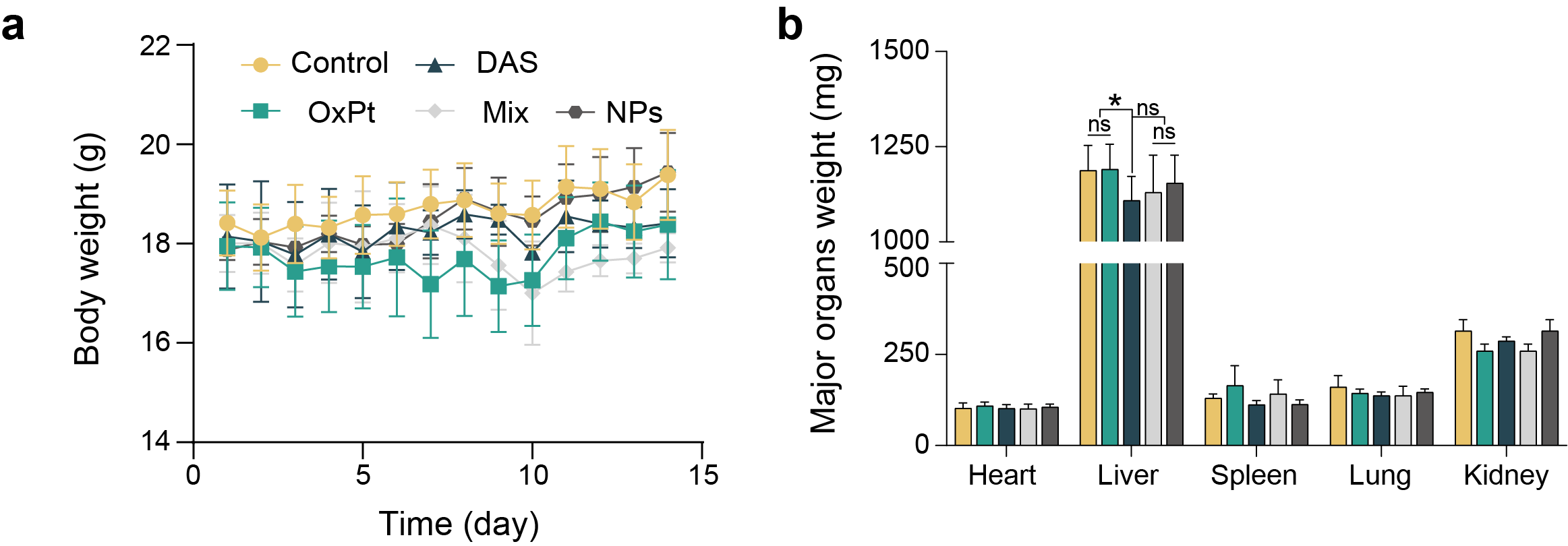


**Figure S19**. (a) Body weights of CT26 tumor-bearing mice during different treatments (n = 5). (b) Major organs of the CT26 tumor-bearing mice were collected and weighed (n = 5).


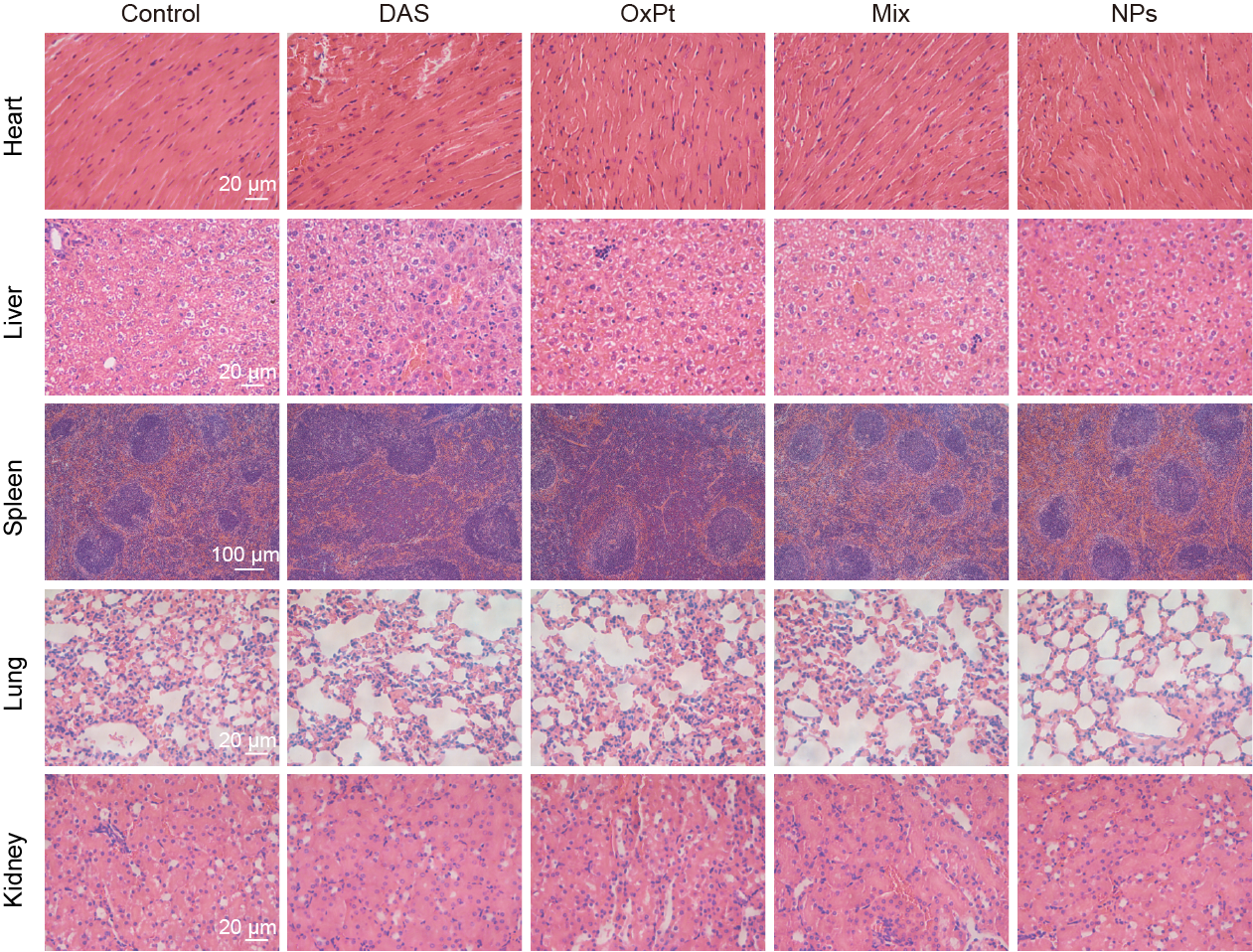


**Figure S20**. The pathological tissue section images of major organs were treated with PBS, DAS, OxPt, Mix, DAS@CD-OxPt (IV) NPs. Scale bars = 100 µm or 20 µm.


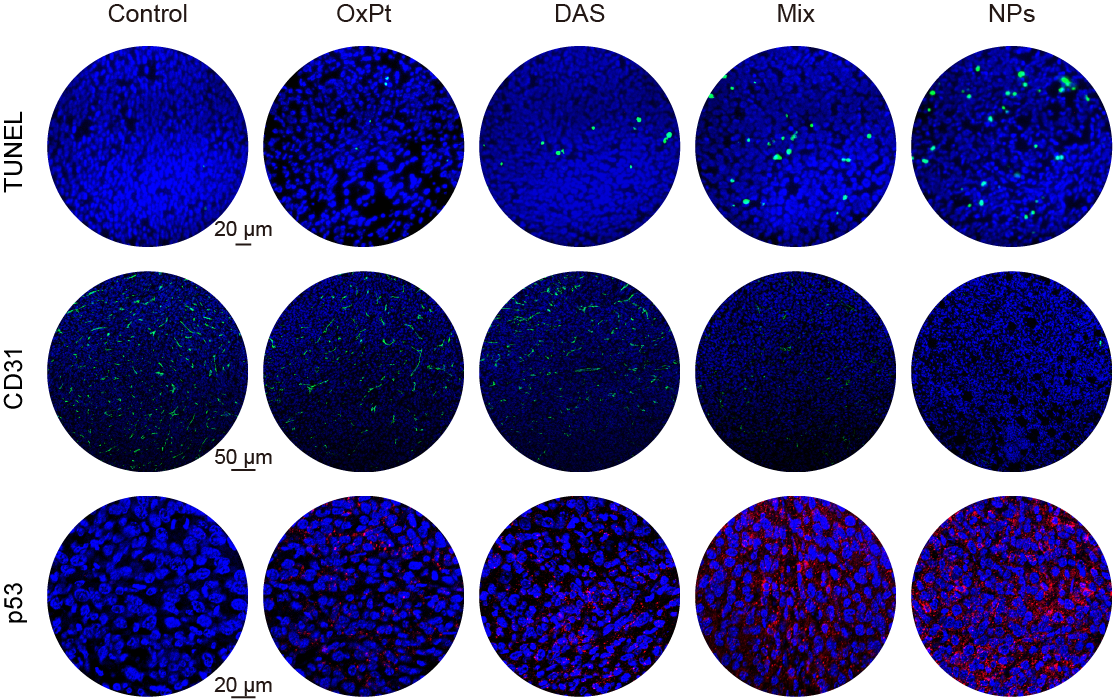


**Figure S21**. Representative TUNEL, CD31, p53 expression images of tumor tissue sections in different groups. Scale bars = 50 µm or 20 µm.


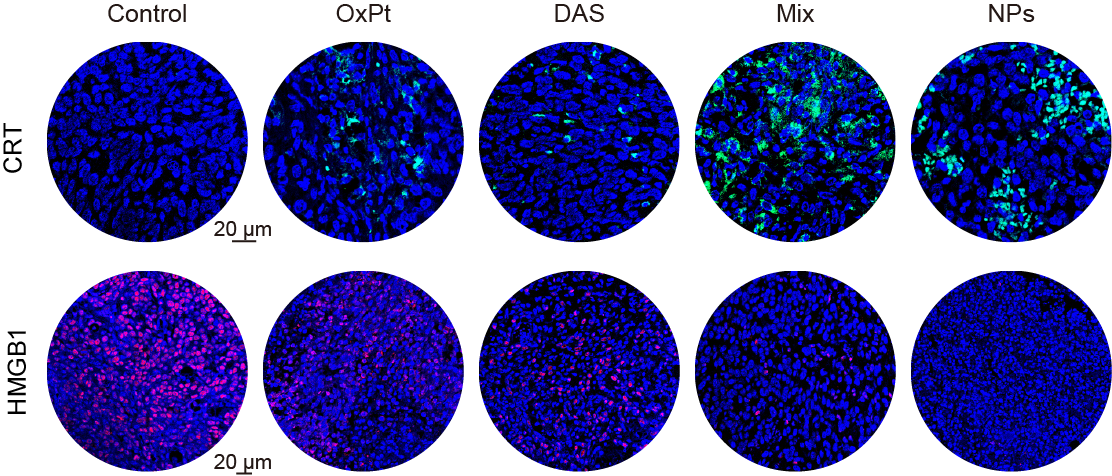


**Figure S22**. Representative CRT, HMGB1 expression images of tumor slices in different groups. Scale bars = 20 µm.


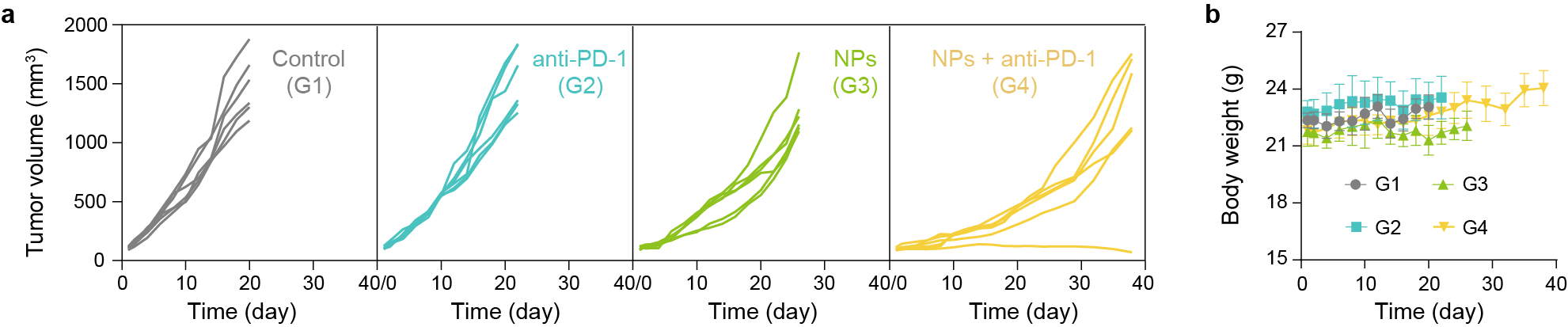


Figure S23. (a) Individual tumor volume of primary tumor after different treatments (G1: Control; G2: anti-PD-1; G3: DAS@CD-OxPt (IV) NPs; G4: DAS@CD-OxPt (IV) NPs + anti-PD-1) (n = 6). (b) Body weight of mice in different treatment groups for survival treatment experiments (n = 6).


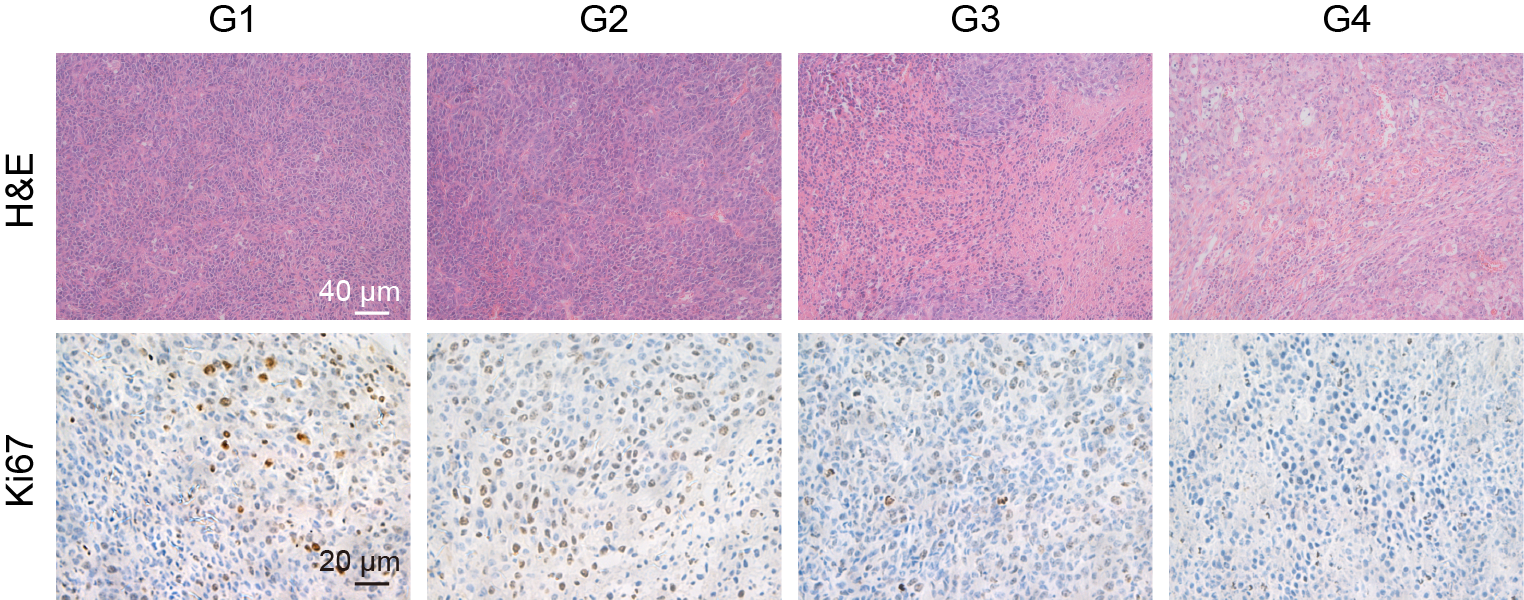


**Figure S24**. The pathological tissue section images of H&E and Ki67 in tumors after different treatments. Scale bars = 40 µm or 20 µm.


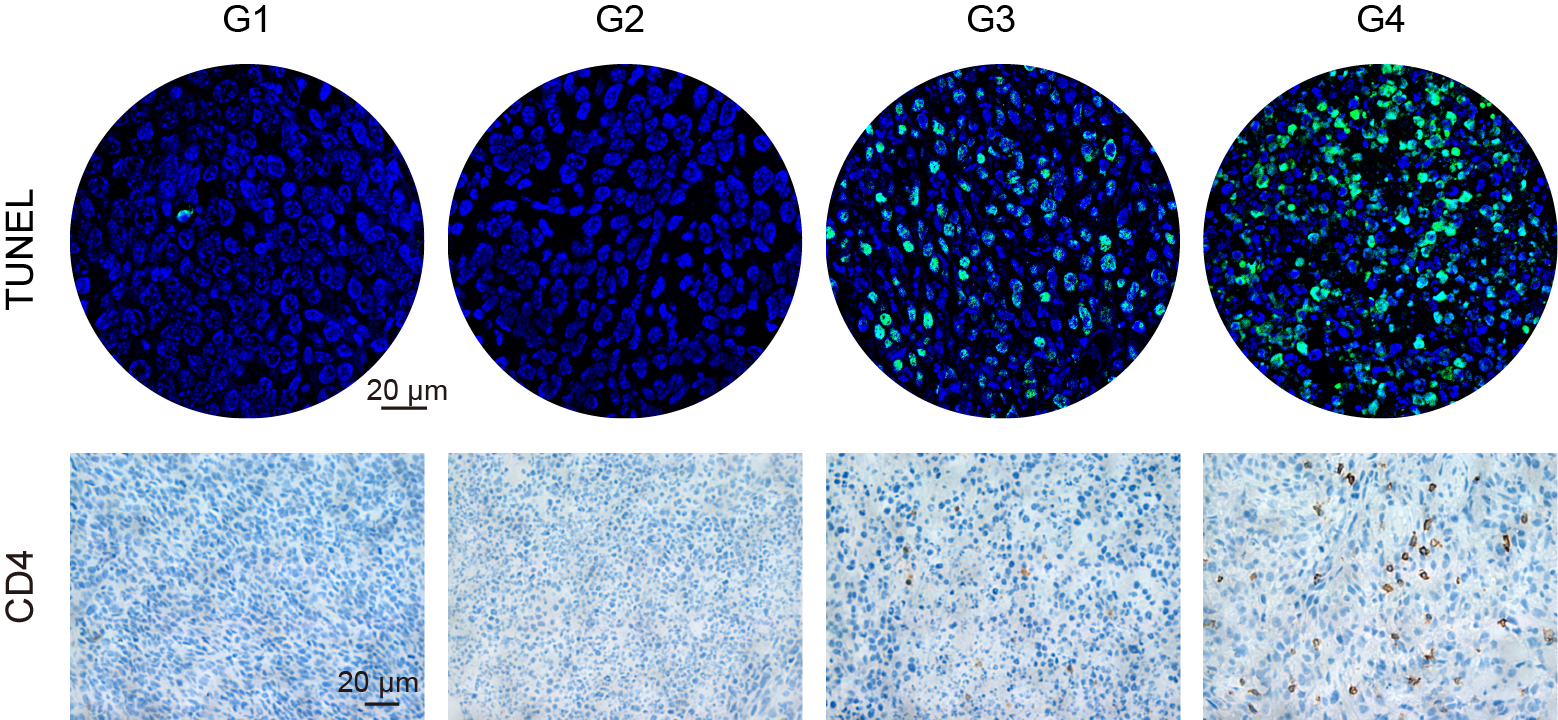


**Figure S25**. Representative TUNEL and immunohistochemical staining of CD4 in different groups. Scale bars = 20 µm.


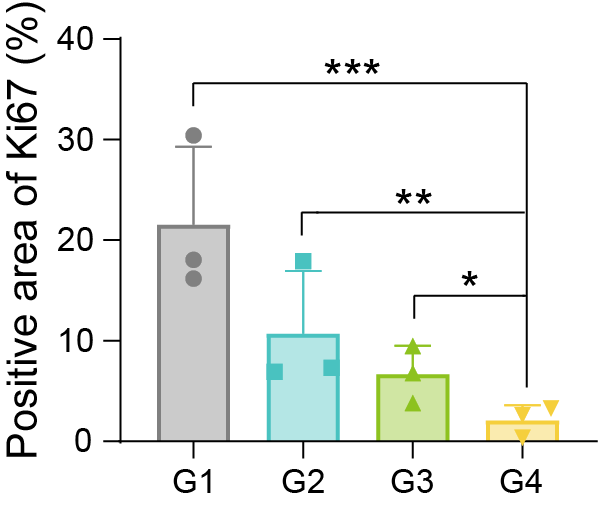


**Figure S26**. Quantitative analysis of Ki67 pathological tissue section image (n = 3).


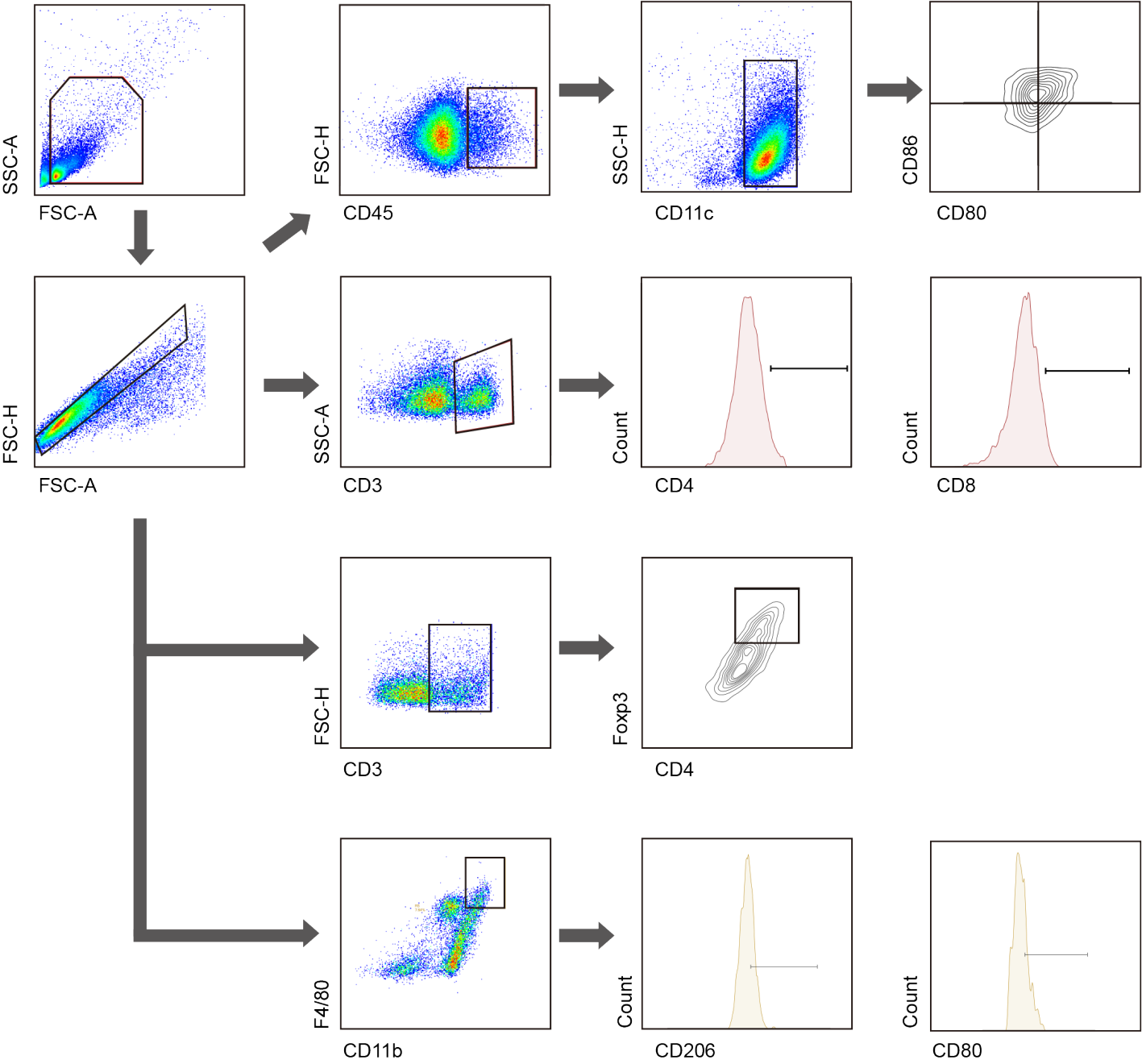


**Figure S27**. Gating strategy of DCs, T cells, Tregs, M1 TAM and M2 TAM.


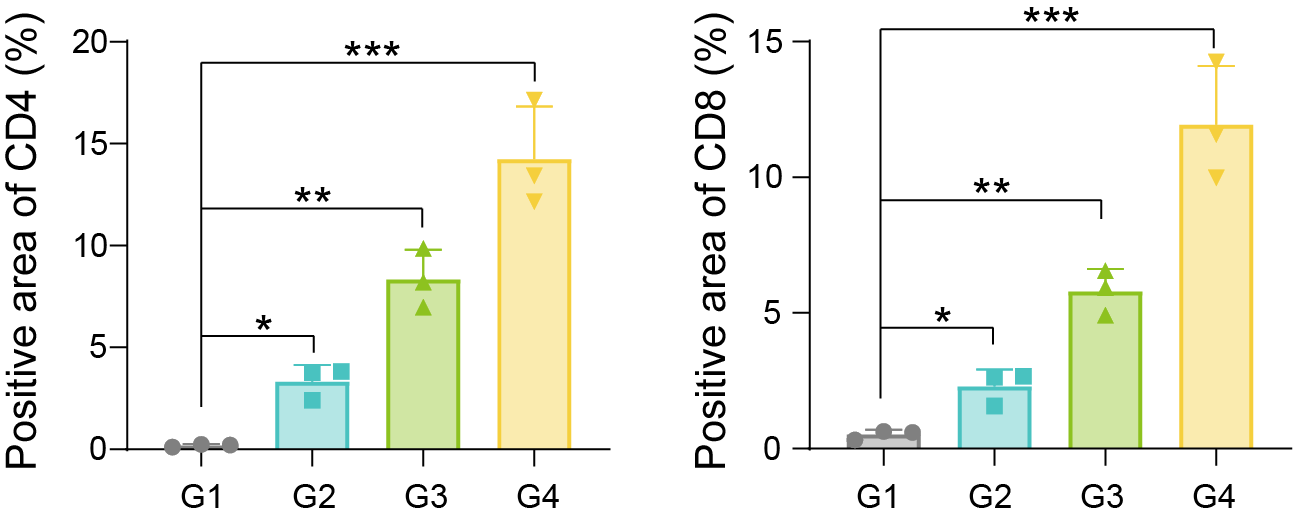


**Figure S28**. Quantitative analysis of CD4^+^ and CD8^+^ immunohistochemical tissue section image (n = 3).


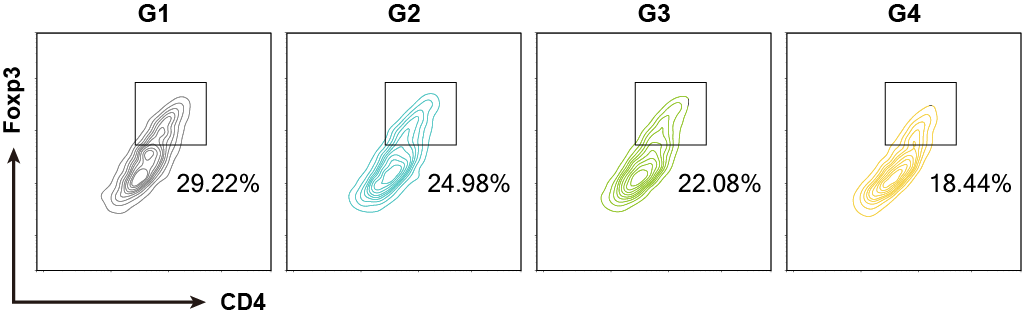


**Figure S29**. Representative flow cytometry plots indicating the proportions of Tregs (in the gate of CD3^+^) in tumors after treatments.


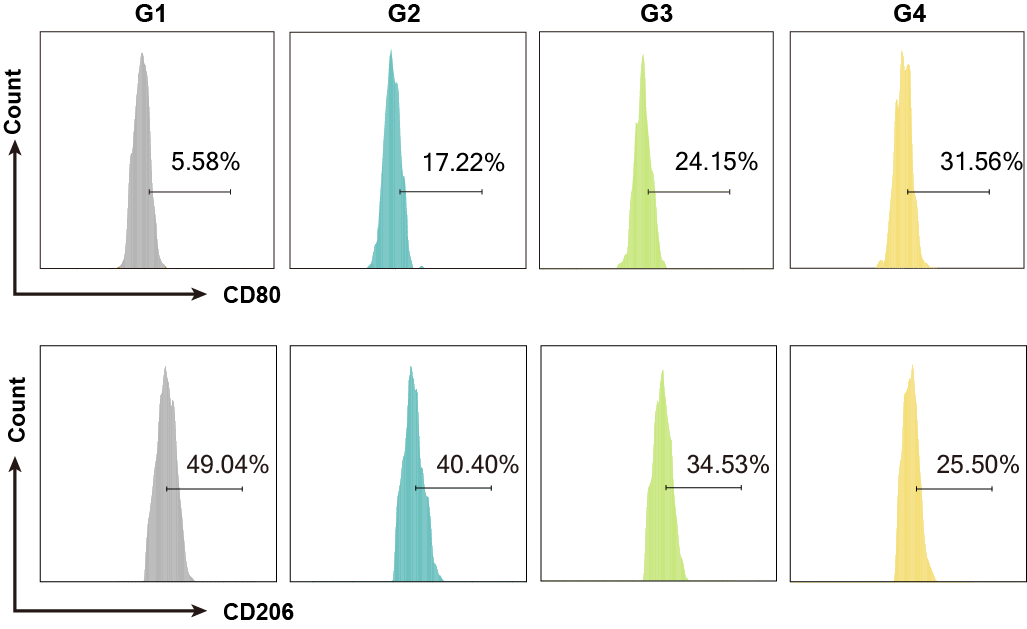


**Figure S30**. Representative flow cytometry plots indicating the proportions of M1-like macrophages (CD80) and M2-like macrophages (CD206) (in the gate of CD11b^+^ F4/80^+^) in tumors after treatments.


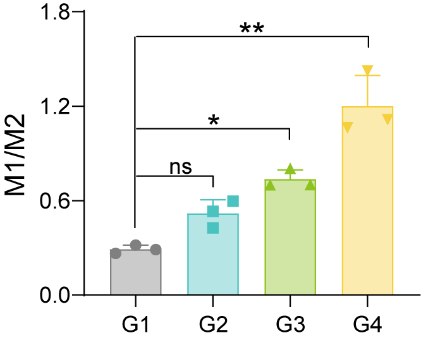


**Figure S31**. Representative flow cytometry plots indicating the proportions of M1-like macrophages (CD80) and M2-like macrophages (CD206) (in the gate of CD11b^+^ F4/80^+^) in tumors after treatments (n = 3).


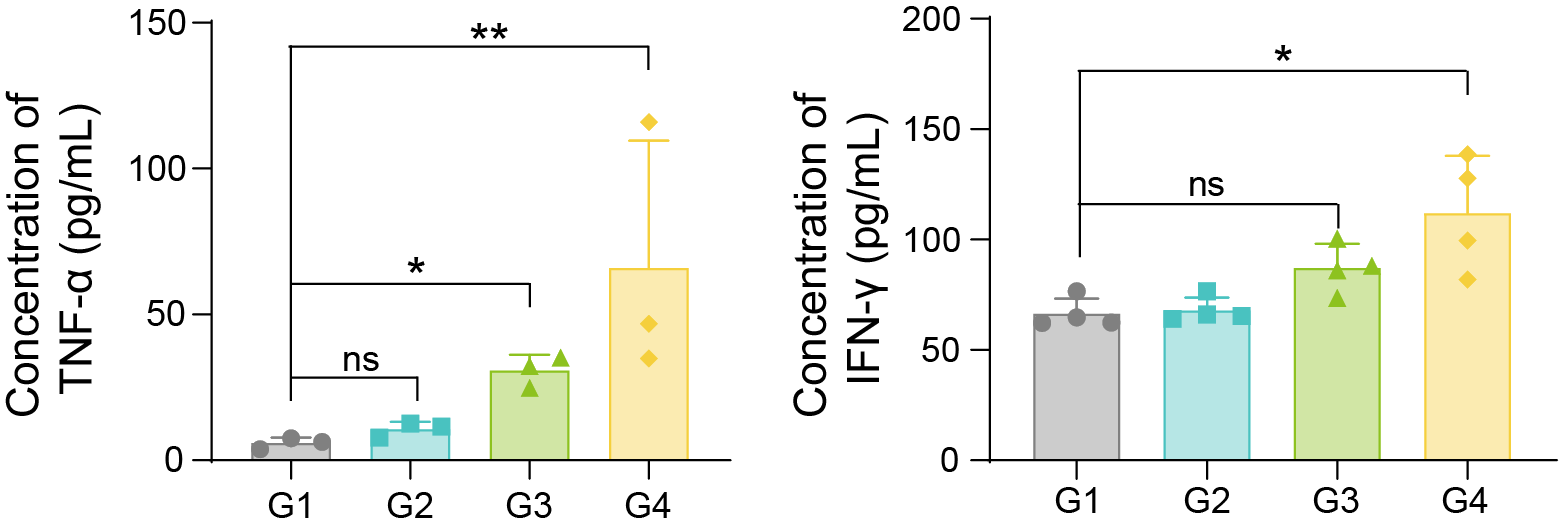


**Figure S32**. The concentration of TNF-α (n = 3) and IFN-γ (n =4) in the serum extracted from the mice after various treatments.


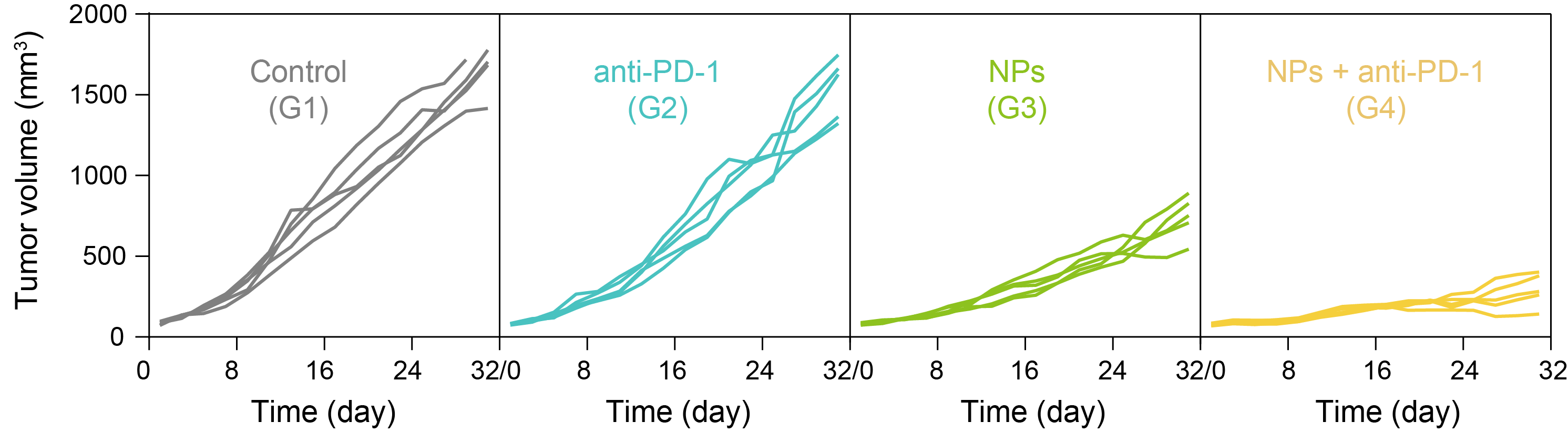


**Figure S33**. Individual tumor volume of lung metastasis model after different treatments (G1: Control; G2: anti-PD-1; G3: DAS@CD-OxPt NPs; G4: DAS@CD-OxPt NPs + anti-PD-1) (n = 5).


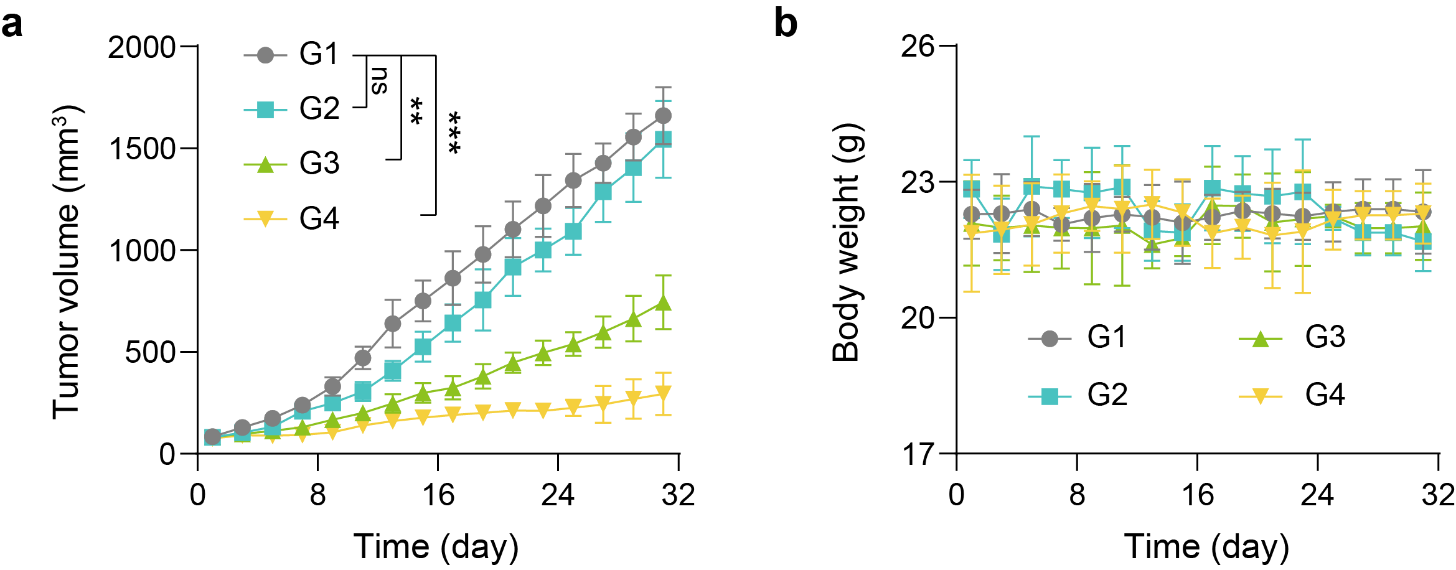


**Figure S34**. (a)The tumor volume and (b) body weight of the lung metastasis model after different treatments (n = 5).


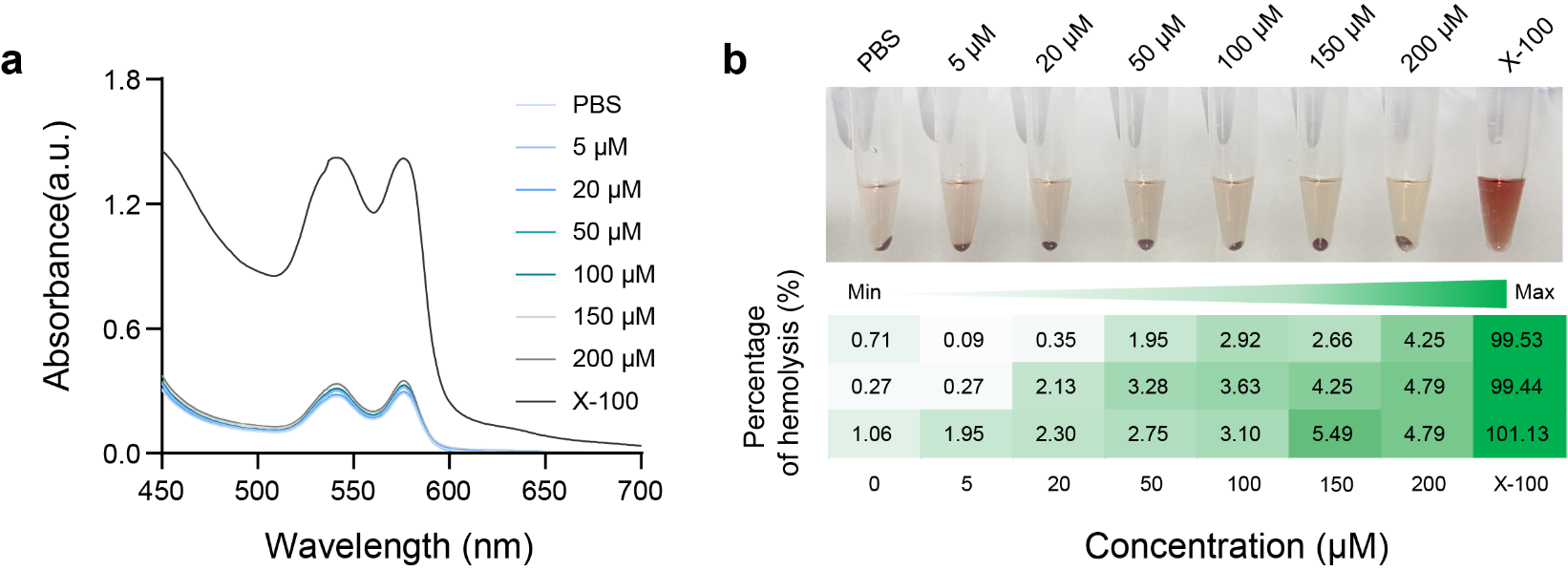


**Figure S35**. UV-vis spectra and a picture of the hemolysis ratio was treated with different concentration of DAS@CD-OxPt NPs (n = 3).


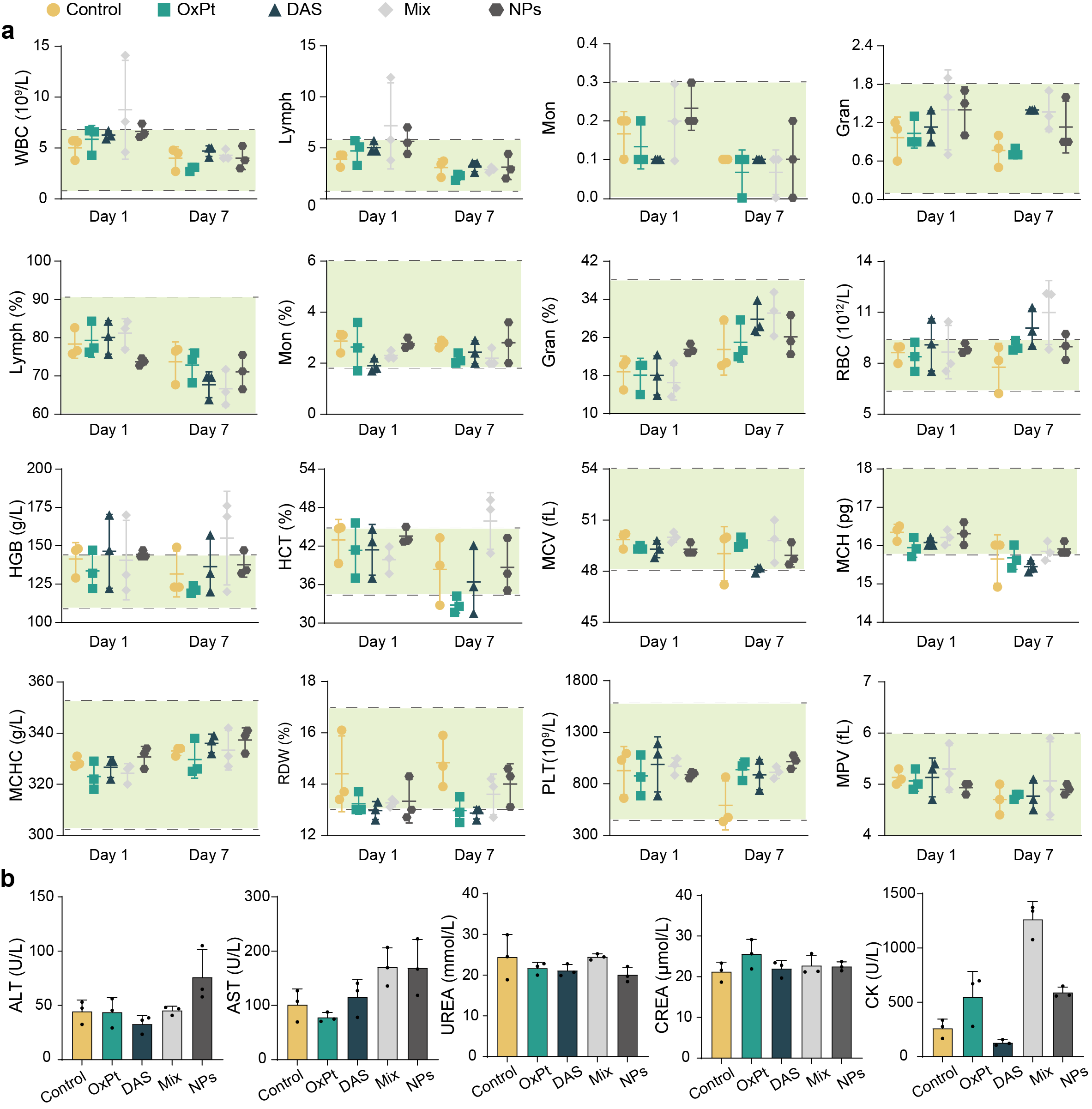


**Figure S36**. Biological safety. a) Blood routine of the Balb/c mice after intravenous administration of drugs in healthy mice, the dashed background box was the normal threshold value of mice (n = 3). b) The alamine aminotransferase (ALT), aspartate aminotransferase (AST), urea (UREA), creatinine (CREA), and creatine kinase (CK) levels of the Balb/c mice after intravenous administration of drugs in healthy mice (n = 3).
